# BRAF^V600E^ augments WNT signaling in colorectal cancer via aberrant DNA methylation

**DOI:** 10.1016/j.isci.2025.112708

**Published:** 2025-05-20

**Authors:** Layla El Bouazzaoui, Jeroen M. Bugter, Emre Küçükköse, André Verheem, Jasmin B. Post, Nicola Fenderico, Inne H.M. Borel Rinkes, Hugo J.G. Snippert, Madelon M. Maurice, Onno Kranenburg

**Affiliations:** 1 Division of Imaging and Cancer, Laboratory Translational Oncology, UMC Utrecht, Utrecht, the Netherlands; 2 Oncode Institute and Center for Molecular Medicine, UMC Utrecht, Utrecht, the Netherlands; 3 Utrecht Platform for Organoid Technology, Utrecht University, Utrecht, the Netherlands

**Keywords:** Epigenetics, Cell biology, Cancer

## Abstract

The BRAF^V600E^ mutation drives an aggressive subtype of colorectal cancer (CRC). Although WNT signaling activation is a hallmark of CRC, APC mutations are uncommon in BRAF-V600E mutant CRC, and RNF43 mutations are instead suspected to drive WNT pathway activation. Here, we investigated WNT pathway activation in BRAF-V600E mutant CRC using CRISPR-LbCpf1-corrected *BRAF* (V600E) and *RNF43* (P441fs) organoids. BRAF^E600V^ organoids regained dependency on EGF receptor signaling, and lost tumorigenic potential. Under identical growth conditions, correction of BRAF^V600E^, rather than RNF43^P441fs^, suppressed WNT target genes and upregulated epithelial differentiation genes and WNT antagonist genes. DNA methylation analysis revealed promoter hypermethylation of WNT antagonist genes and gene body hypermethylation —associated with transcriptional upregulation— of key WNT effectors (LGR5, EPHB2, and TCF4) in BRAF^V600E^ organoids. Demethylation treatment resulted in upregulation of WNT antagonists and reduced WNT target gene expression in BRAF^V600E^ organoids. Our results demonstrate that BRAF^V600E^ enhances WNT pathway activation through modulation of DNA methylation patterns.

## Highlights

• CRISPR-mediated genome editing reveals late-stage cancer cell reliance on driver mutations
• Unexpectedly, the BRAF^V600E^ mutation mediates increased WNT target gene expression
• BRAF^V600E^ augments WNT signaling by altering the DNA methylation landscape
• DNA demethylating agents are effective WNT signaling inhibitors in BRAF-V600E mutant CRC

## Graphical abstract

**Figure:**
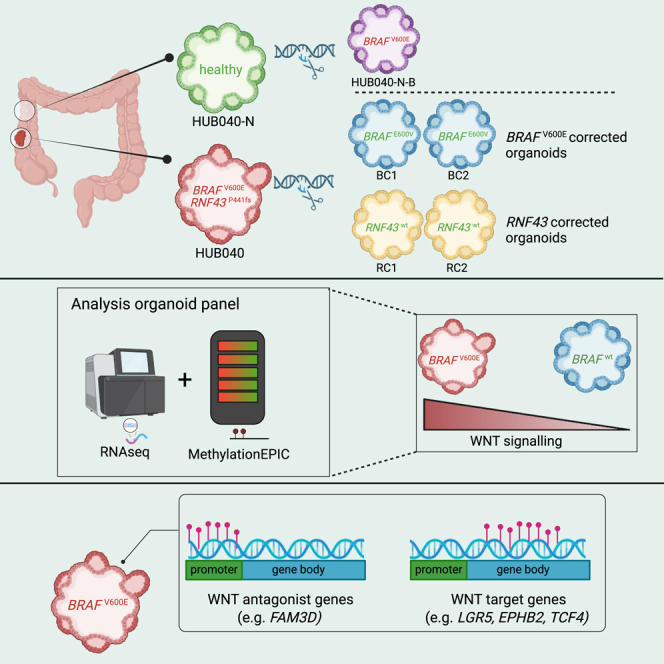

## Introduction

BRAF^V600E^ mutations are found in approximately 10% of all cases of colorectal cancer (CRC),^1^ and promote cell proliferation and survival by RAS-independent mitogen-activated protein kinase (MAPK) pathway activation. The vast majority of BRAF-V600E mutant CRCs belong to a specific subtype that is characterized by microsatellite instability (MSI), and the CpG island methylator phenotype (CIMP).^2^^,^^3^ This subtype of CRC is associated with poor prognosis in the metastatic setting.^4^^,^^5^

A striking feature of BRAF-V600E mutant CRCs is the low frequency of inactivating mutations in the *APC* tumor suppressor gene, which drives activation of WNT signaling in the vast majority of BRAF wildtype sporadic CRCs. Rather, BRAF^V600E^ mutations frequently co-occur with mutations in RNF43,^6^^,^^7^ a negative regulator of WNT ligand receptors.^8^ It is presumed that loss-of-function mutations in RNF43 cause accumulation of WNT receptors on the plasma membrane and consequent hypersensitivity to WNT ligands.^8^^,^^9^ Interestingly, of the truncating mutations in RNF43 frequently occurring in CRC, only a fraction results in hyperactivation of WNT signaling.^10^ It is currently unknown whether truncating mutations in RNF43 are sufficient to achieve levels of WNT pathway activation that cause oncogenic cellular transformation.

Most studies on the role of the BRAF^V600E^ mutation in CRC have used “forward” approaches, either by overexpressing mutant BRAF or by introducing the V600E mutation in normal epithelial cells.^11^^,^^12^^,^^13^^,^^14^ However, this approach is less suited to study the continued dependency of advanced cancers —harboring many additional (epi-)genetic alterations— on the BRAF^V600E^ mutation.

Here, we used aggressive colon cancer patient-derived organoids (PDO) with co-occurring mutations in BRAF (V600E) and RNF43 (P441fs). We applied a reverse CRISPR approach to correct these mutations and study the subsequent changes in tumor biology. Unexpectedly, we found that the BRAF^V600E^ mutation, rather than RNF43^P441fs^, was essential for maintaining high levels of WNT signaling and tumorigenic capacity. BRAF^V600E^ represses expression of multiple WNT antagonist genes and stimulates WNT signaling by inducing DNA methylation of the promoters of negative WNT regulators (leading to reduced expression), and of the gene bodies of WNT target genes and of key WNT-effector gene *TCF4* (associated with increased expression^15^^,^^16^^,^^17^^,^^18^^,^^19^^,^^20^). Pharmacological DNA demethylation was sufficient to suppress WNT target gene expression in BRAF^V600E^ PDOs. The results delineate a key role for BRAF^V600E^ in the induction of cancer-associated high WNT signaling.

## Results

### Correction of the V600E mutation in BRAF in colon cancer organoids derived from an advanced tumor

To study the role of the BRAF^V600E^ mutation in the context of advanced colon cancer, we used a PDO from a stage IV tumor (HUB040). Whole genome sequencing revealed that this PDO contains multiple pathogenic cancer gene census mutations, including a frameshift (truncating) mutation in the *RNF43* gene (P441fs), potentially driving WNT signaling^10^ (Figure S1A). Moreover, HUB040 contains multiple chromosomal copy number changes characteristic of CRC, consistent with chromosomal instability (Figure S1B).

**Figure 1:**
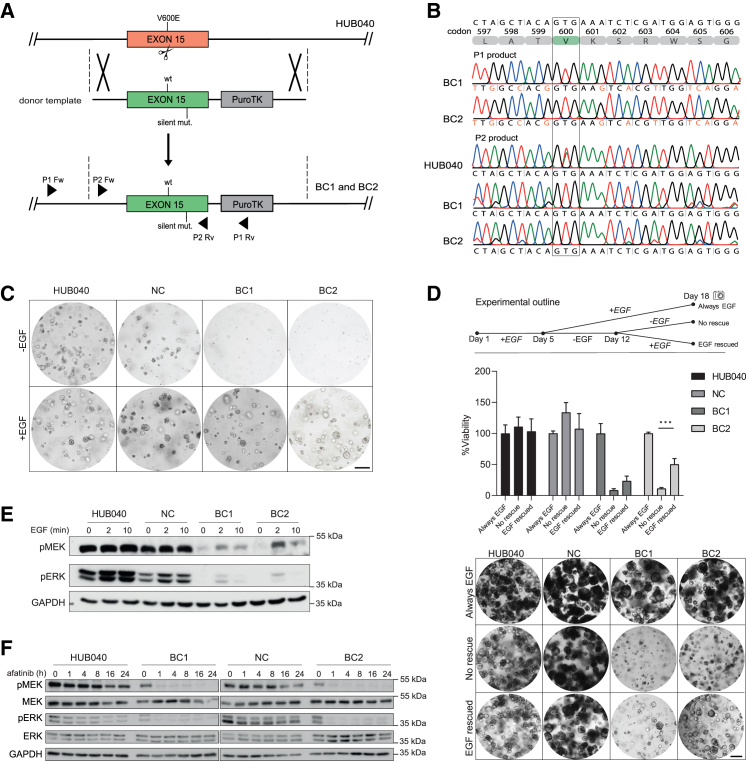
Correction of the BRAF-V600E mutation leads to renewed growth dependency on EGF (A) Schematic representation of the gene-targeting strategy for correcting the *BRAF*-V600E (T/A) point mutation in the CRC PDO HUB040 using the CRISPR-LbCpf1 system. Scissors indicate the double-strand break (DSB) generated by the V600E-specific sgRNA. A donor vector that contains the entire wildtype exon 15 of the *BRAF* gene, including silent mutations, and a PuroTK selection cassette, flanked at both ends by the homology arms was used as a template to induce the homology-directed repair (HDR) mechanism after the CRISPR-LbCpf1-mediated DSB. Primer pair 1 was designed outside of the left homology arm to prevent detection of the donor plasmid. Primer pair 2 was designed to validate complete elimination of the mutant sequence. (B) Identification of BRAF-V600E corrected organoids using PCR analysis. Sanger sequence results from two independent organoids (BC1 and BC2) indicate precise integration of the desired sequence into the genome. Introduced silent mutations are marked in orange. Primer pair 2 products indicate that the mutant *BRAF*-V600E sequence was successfully eliminated in the BRAF-V600E corrected organoids, while the mutant sequence was still detected in the original organoids line HUB040. (C) BC1 and BC2 grow exclusively in the presence of EGF. Organoids were grown for 9 days after dissociating them into single cells in organoid growth medium with or without EGF. Scale bar = 250 μm. (D) HUB040, NC, BC1, and BC2 were grown in CRC organoid medium supplemented with EGF for 5 days after which organoids were starved for 7 consecutive days from EGF. On day 12, organoids were either rescued with CRC medium supplemented with EGF or starved from EGF for 6 more days. Organoid viability was measured with CellTiter-Glo 3D assays and viability was compared to organoids that received EGF supplemented medium for 18 consecutive days without a starvation period. Organoid pictures were taken on day 18. Data are presented as mean ± SD. Scale bar = 250 μm. (E) Immunoblot analysis of HUB040, NC, BC1, and BC2 starved overnight from essential medium supplements and subsequently stimulated with 50 ng/mL EGF for 2 or 10 min. (F) Immunoblot analysis of HUB040, NC, BC1, and BC2 treated with EGFR-inhibitor afatinib for indicated hours. Unpaired *t*-test ∗∗∗*p* < 0.001.

The CRISPR-LbCpf1 system was used to correct V600E in *BRAF* back to the wildtype sequence E600V (Figure 1A). As correction of the mutant *BRAF*^V600E^ allele may cause a renewed dependency on EGF,^21^ the clone selection medium was supplemented with EGF immediately after transfection to prevent the potential loss of successfully corrected organoids. Individual organoids that survived selection were manually picked and clonally expanded and analyzed by Sanger sequencing (Figure 1B), and BRAF V600 droplet digital PCR (ddPCR) (Figure S2). Successful correction of the BRAF^V600E^ mutation was demonstrated in two independently established organoids (BRAF-Corrected-1 (BC1) and BC2). A non-corrected organoid (NC) that underwent the same selection procedure as the BC organoids was also included for further analyses. Correction of the RNF43^P441fs^ mutation in HUB040 has been described previously,^22^ and also resulted in two independently established organoids (RNF43-Corrected-1 (RC1) and RC2). Also, here, an NC that underwent the same selection procedure as the RC organoids was included.

### Correction of the V600E mutation in BRAF leads to renewed growth dependency on EGF

To test the growth dependency of successfully corrected organoids on exogenously added EGF, single cells of BRAF^V600E^ (HUB040 and NC) and BRAF^E600V^ organoids (BC1 and BC2) were seeded in growth medium supplemented with or without EGF. While HUB040 and NC thrived in media with or without EGF, BC1 and BC2 only grew in medium supplemented with EGF (Figure 1C). We next tested whether BC1 and BC2 could still be rescued with EGF supplementation following EGF depletion. HUB040, NC, BC1, and BC2 organoids were initially cultured in CRC organoid medium supplemented with EGF for 5 days following splitting. Subsequently, the organoids underwent a 7-day starvation period devoid of EGF. On day 12, the organoids were subjected to one of two conditions: either rescued with CRC medium supplemented with EGF or continued EGF deprivation for an additional 6 days. Re-supplementation of EGF after starvation stimulated re-growth of BC1 and BC2, although not to the extent of HUB040 and NC (Figure 1D).

### Correction of the V600E mutation in BRAF leads to restoration of transient EGF-dependent ERK pathway activation

Next, the effect of V600E correction on basal and EGF-induced activation of the MEK/ERK pathway was assessed. HUB040, NC, BC1, and BC2 organoids were starved for 16 h from growth factors. HUB040 and NC retained very high basal levels of phosphorylated MEK (pMEK) and ERK (pERK) in the absence of growth factors (Figure 1E), and this was further increased following stimulation with EGF, in line with previous findings.^23^ By contrast, growth factor-starved BC1 and BC2 had very low pMEK and pERK levels. Stimulation with EGF caused a transient increase in pMEK and pERK levels, as is commonly observed in non-transformed cells.^24^^,^^25^ However, the level of transient ERK pathway activation following EGF stimulation was still much lower than the level of constitutive ERK pathway activation caused by the BRAF^V600E^ mutation. Treatment of actively growing organoids with the EGFR-inhibitor afatinib caused a strong reduction of the pMEK and pERK signals to undetectable levels in BC1 and BC2, while afatinib minimally affected pMEK and pERK levels in HUB040 and NC (Figure 1F).

### The V600E mutation in BRAF is indispensable for tumor-initiating capacity

To test how correction of the BRAF^V600E^ mutation affects tumor-initiating capacity, immunodeficient mice were subcutaneously injected with HUB040 and NC or BC1 and BC2. While mice injected with either HUB040 (2/3; 66%) or NC (4/5; 80%) developed tumors, those injected with BC1 (0/5; 0%) or BC2 (0/5; 0%) did not (Figure S3).

### Correction of the V600E mutation in BRAF leads to suppression of WNT signaling and concomitant differentiation

The above results indicate that correction of a single mutation (V600E in BRAF) in late-stage cancer cells, causes major changes in tumor biology. To gain insight into the underlying mechanisms, we assessed how correction of the BRAF^V600E^ mutation alters the cellular transcriptome. To this end, RNA sequencing was performed of HUB040 and NC versus BC1 and BC2 organoids grown in the presence or absence of EGF (Figure S4A). PCA plot analysis showed that BRAF status (V600 versus E600) was the most dominant cause of transcriptional variation, with little contribution by culture conditions (Figure S4B). Differential gene expression analysis was conducted by grouping HUB040 and NC across all culture conditions and comparing them to BC1 and BC2, also across all culture conditions.

**Figure 2:**
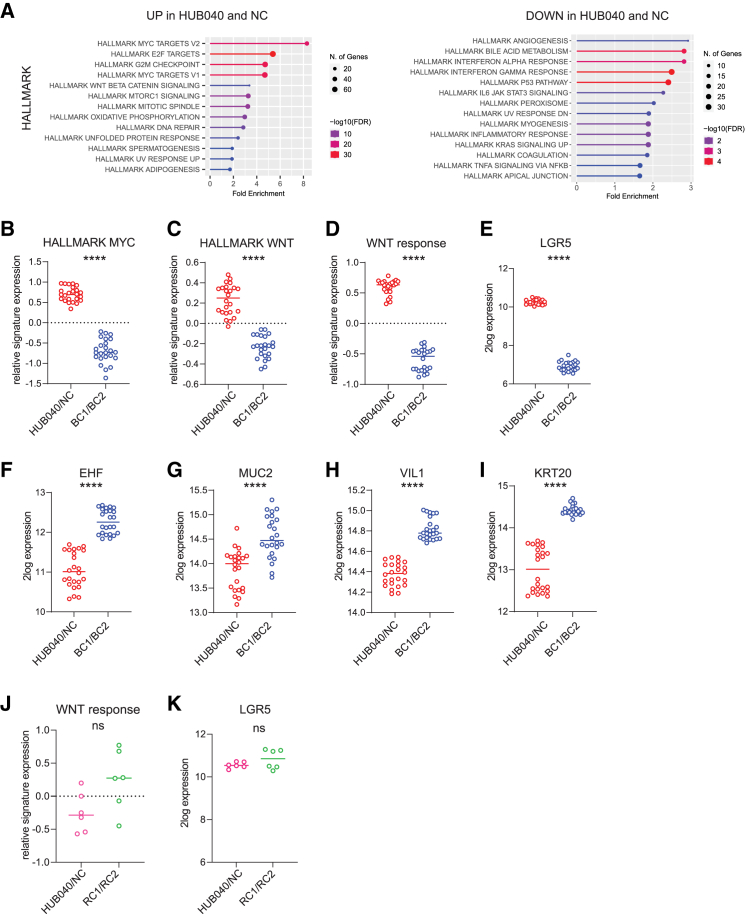
Correction of the V600E mutation in BRAF leads to suppression of WNT signaling and concomitant differentiation (A) Enrichment analysis of transcripts that were significantly differentially expressed between HUB040 and NC vs. BC1 and BC2 (|log2 fold-change| >0.5; *p* value < e−10), using the MSigDB Hallmark genesets. Gene enrichment analysis was performed using ShinyGO. Relative signature expression (z-scores) of (B) Hallmark MYC, (C) Hallmark WNT, and (D) WNT target gene signature (Van Der Flier et al.) in HUB040 and NC vs. BC1 and BC2. 2log expression of (E) LGR5, (F) EHF, (G) MUC2, (H) VIL1, and (I) KRT20. (J) Relative signature expression (z-scores) of WNT response in RNF43 mutant organoids HUB040 and NC vs. RNF43 corrected organoids RC1 and RC2. (K) 2log expression of LGR5 in HUB040 and NC vs. RC1 and RC2. Unpaired *t*-test ns = nonsignificant, ∗∗∗∗*p* < 0.0001. FDR, false discovery rate.

Indeed, we identified 3911 differentially expressed genes (P < e^−10^; |log2 fold-change| > 0.5) between HUB040 and NC organoids and BC1 and BC2 organoids. Of these, 1788 genes were upregulated and 2123 were downregulated in HUB040 and NC (Table S1). Analysis of the expression of Hallmark, KEGG, and Gene Ontology (GO) Biological Processes pathway gene sets, revealed that multiple aspects of tumor biology were changed following correction of the BRAF^V600E^ mutation, including proliferation, cell growth, and metabolism (Figure 2A and S4C). MYC and WNT pathways were significantly upregulated in HUB040 and NC (Figures 2B, 2C, and S5). A WNT response signature^26^ consisting of genes induced by WNT signaling, and LGR5 (a WNT-driven intestinal stem cell marker) were strongly reduced in BC1 and BC2 (Figures 2D, 2E, and S6). Conversely, key markers of intestinal stem cell differentiation, such as EHF, MUC2, VIL1, and KRT20, were strongly upregulated in BC1 and BC2 (Figures 2F–2I).

To further validate these findings, we repeated the RNA-seq experiment under stricter EGF starvation conditions, where in addition to EGF depletion, all additive growth factors in the organoid medium were also depleted. This revealed a more pronounced effect of EGF depletion at a transcriptional level (Figure S7A). However, the transcriptional differences observed remained consistent across both experimental conditions (Figures S7B–S7D). Moreover, expression of the proliferation marker KI67 and immediate-early genes (IEGs)^27^ were comparable between *BRAF*-V600E mutant and corrected organoids (Figures S8A–S8C), confirming that the observed changes in WNT signaling are a direct consequence of V600E mutation correction rather than an indirect consequence of reduced IEG expression or proliferation. Thus, BRAF^V600E^ correction decreased WNT signaling activity, despite the presence of mutant RNF43, a known WNT tumor suppressor. In contrast, when BRAF^V600E^ organoids were treated with the BRAF inhibitor encorafenib, transcriptomic analysis revealed no significant changes in WNT signaling activation (Figure S9), likely as a result of feedback mechanisms. Titration of WNT ligands into the medium revealed no consistent differences associated with the BRAF^V600E^ mutation (Figure S10A). Similarly, treatment with the porcupine inhibitor LGK-974 showed no significant differences in sensitivity between BRAF^V600E^ and BRAF^E600V^ organoids (Figure S10B), indicating that correction of the V600E mutation does not alter WNT ligand dependency.

Interestingly, correction of the RNF43^P441fs^ mutation itself, which may affect WNT hyperactivation in BRAF/RNF43 mutant tumors,^10^ did not result in an altered expression of WNT target genes, including LGR5 (Figures 2J and 2K). However, it is important to note that RNF43^P441fs^ corrected organoids required supplementation with both WNT and RSPO in the growth medium, conditions that may mask effects of the RNF43 mutation in WNT hyperactivation. Of note, the RNF43 mutation was found essential for maintaining the mucinous differentiation phenotype and for metastatic, but not tumorigenic capacity.^22^

### Correction of the V600E mutation in BRAF leads to upregulation of WNT antagonist genes

**Figure 3:**
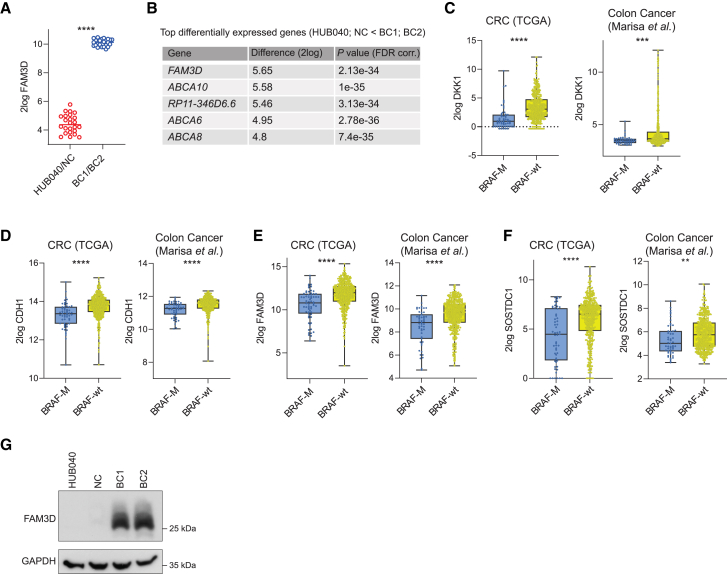
Correction of the V600E mutation in BRAF leads to upregulation of WNT antagonist genes mRNA expression (2log) of WNT antagonist (A) FAM3D in HUB040 and NC versus BC1 and BC2. (B) Top differentially expressed genes (HUB040 and NC < BC1 and BC2) sorted on 2log difference (*t*-test, FDR corrected). mRNA expression of (C) DKK1, (D) CDH1, (E) FAM3D, and (F) SOSTDC1 in CRC TCGA dataset (BRAF-M, *n* = 57; BRAF-wt *n* = 467), and colon cancer dataset Marisa et al. (BRAF-M, *n* = 51; BRAF-wt *n* = 461). (G) Immunoblot analysis of FAM3D in HUB040, NC, BC1 and BC2. Unpaired t-test ∗∗*p* < 0.01, ∗∗∗*p* < 0.001, ∗∗∗∗*p* < 0.0001. CRC, colorectal cancer; TCGA, The Cancer Genome Atlas.

The above data indicate that the BRAF^V600E^ mutation is a major contributor to WNT pathway activation in HUB040 organoids. WNT pathway activation can also be achieved by promoter hypermethylation of WNT antagonist genes.^28^ Analysis of the RNA-seq data showed that multiple WNT antagonist genes (16/135; GO_0090090) were expressed to significantly lower levels in HUB040 and NC than in BC1 and BC2 (Table 1), including *DKK1* and *CDH1*. Another WNT antagonist gene, *FAM3D*,^29^ was the most strongly upregulated gene following V600E correction (Figures 3A and 3B). Analysis of large publicly available CRC datasets^30^^,^^31^ revealed that of the 17 BRAF-controlled WNT antagonist genes only *DKK1*, *CDH1*, *SOSTDC1*, and *FAM3D* were consistently expressed to significantly lower levels in BRAF*-*mutant CRCs versus BRAF-wildtype CRCs (Figures 3C–3F, S11, and S12). Among these, FAM3D displayed strongly decreased protein levels in HUB040 and NC versus BC1 and BC2 (Figure 3F).

**Table 1:** Downregulated negative regulators of canonical WNT signaling in *BRAF*^V600E^ organoids

Gene^a^	*p* value (corr.)	Difference (2log)
*CDH1*	2.02E-35	1.084955
*PTPRU*	6.77E-27	0.987819
*SCYL2*	2.60E-20	0.719623
*SOSTDC1*	1.75E-18	1.831014
*ROR2*	2.03E-18	1.808907
*DKK1*	1.30E-16	2.609147
*FRMD8*	1.54E-15	0.962649
*FOXO1*	1.90E-15	0.748777
*CAV1*	2.34E-13	1.178518
*DDIT3*	4.32E-13	0.990509
*MCC*	5.70E-13	1.161701
*CTNND1*	9.02E-13	0.638719
*SNAI2*	1.03E-12	1.193397
*INVS*	1.38E-12	0.669499
*SLC9A3R1*	2.18E-12	0.577392
*TMEM88*	1.96E-11	1.060651

a Gene Ontology Biological processes, GO_0090090.

### BRAF^V600E^ CRC organoids show promoter hypermethylation of *FAM3D* and *CTNND1*

**Figure 4:**
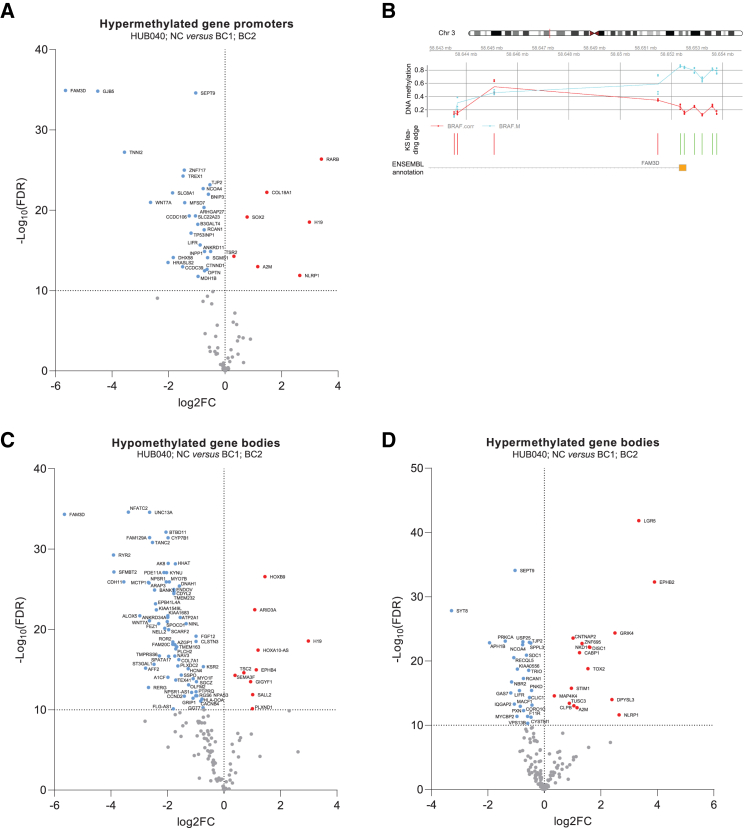
Correction of the BRAF-V600E mutation alters the DNA methylation landscape (A) Volcano plot of mRNA expression data of hypermethylated gene promoter annotated genes in HUB040 and NC versus BC1 and BC2. (B) Gene promoter methylation of *FAM3D*. Methylation is quantified with beta values. Each point represents the methylation of each sample and lines link the mean methylation of each group. KS leading edge panel marks with green bars CpGs contributing to the enrichment score and with red the rest of them. (C) Volcano plot of mRNA expression data of hypomethylated gene body annotated genes in HUB040 and NC versus BC1 and BC2. (D) Volcano plot of mRNA expression data of hypermethylated gene body annotated genes in HUB040 and NC versus BC1 and BC2. FC, fold change; FDR, false discovery rate; KS, Kolmogorov–Smirnov.

The BRAF^V600E^ mutation induces genome-wide DNA methylation changes.^13^^,^^32^ In addition, WNT antagonist genes are subject to silencing by DNA methylation.^28^^,^^33^ Therefore, we hypothesized that DNA methylation may contribute to suppression of WNT pathway antagonists in BRAF^V600E^ organoids. To test this, the Infinium MethylationEPIC assay was used to identify differentially methylated regions (DMRs) residing within gene promotors and gene bodies comprising >5 CpG sites. PCA plot analysis showed that BRAF mutation status was the major cause underlying DNA methylation differences (Figure S13). Within promoter regions, 87 hypermethylated DMRs and 214 hypomethylated DMRs were identified in HUB040 and NC versus BC1 and BC2 (FDR <0.01) (Table S2). Of the genes annotated to hypermethylated gene promoters in HUB040 and NC, 48% showed low mRNA levels in HUB040 and NC (vs. BC1 and BC2) (*p* < 0.01) (Figure 4A). Of the genes annotated to hypomethylated gene promoters in HUB040 and NC (vs. BC1 and BC2) 37% were transcriptionally upregulated (*p* < 0.01) (Figure S14).

The gene promoter with the largest methylation difference (HUB040 and NC > BC1 and BC2) was the *FAM3D* gene promoter (Table S2; Figure 4B). Next to *FAM3D*, promoter hypermethylation of the WNT antagonist gene *CTNND1* was also observed in HUB040 and NC (Table S2). Promoter hypermethylation of *CDH1* and *DKK1*, and the other transcriptionally downregulated WNT antagonist genes, was not observed, suggesting another mode of gene repression initiated by the BRAF^V600E^ mutation.

Another noteworthy observation was that the gene promoter with the most significant methylation difference (HUB040 and NC > BC1 and BC2) (Table S2) was the *SEPT9* gene promoter. Methylated SEPT9 DNA is an FDA-approved screening biomarker in CRC,^34^ which has also been found to be associated with BRAF mutations.^35^

### BRAF^V600E^ CRC organoids show gene body hypermethylation of *LGR5* and *EPHB2*

While DNA methylation of gene promoters is a repressive epigenetic mark, DNA methylation within gene bodies is associated with higher levels of gene transcription.^15^^,^^16^^,^^17^^,^^18^^,^^19^^,^^20^ DMRs within gene bodies were assessed, where we found 186 hypomethylated and 194 hypermethylated gene bodies in HUB040 and NC (vs. BC1 and BC2) (Table S3). Of the genes annotated to hypomethylated gene bodies in HUB040 and NC 82% were concordantly transcriptionally downregulated (*p* < 0.01) (Figure 4C). Further analyses were limited to gene body methylation changes in WNT signaling genes. Interestingly, a hypomethylated DMR within the gene body of *FAM3D* (*p*=1.2e-5) was found. Gene body hypomethylation of negative WNT regulator genes *ROR2*,^36^ and *CDH11*,^37^ both transcriptionally downregulated in HUB040 and NC, was also observed.

Of the genes annotated to hypermethylated gene bodies in HUB040 and NC (vs. BC1 and BC2), 25% of genes were transcriptionally upregulated (*p* < 0.01) in HUB040 and NC (Figure 4D). Interestingly, both *LGR5* and *EPHB2* had extensive gene body hypermethylation (Figures S15A and S15B). In addition, both genes had significantly higher transcript levels in HUB040 and NC vs. BC1 and BC2 (Figures 4D; Table S1). Of note, the gene body of *TCF4*, a key transcriptional activator of canonical WNT signaling, was hypermethylated in HUB040 and NC, with also significantly higher transcript levels in HUB040 and NC vs. BC1 and BC2 (Table S3; Figure S16). Gene body hypermethylation of *TCF4* and WNT-target genes (including *LGR5* and *EPHB2*) is, therefore, likely to contribute to high WNT signaling in BRAF-V600E mutant CRCs.

### The V600E mutation in BRAF promotes WNT signaling activation in healthy colon organoids

The dependency of high WNT signaling on the BRAF^V600E^ mutation in late-stage tumor cells raises the question whether the mutation acts independently to promote WNT signaling or whether it amplifies WNT signaling in combination with other (epi-)genetic aberrations in late-stage cancer cells. To test this, the BRAF^V600E^ mutation was introduced into healthy colon organoids derived from the same patient (HUB040-N), using CRISPR technology (Figure S17A). Successful introduction of the BRAF^V600E^ mutation in HUB040-N (HUB040-N-B) was confirmed by PCR analysis and Sanger sequencing (Figures S17B and S17C). Introduction of the V600E mutation caused constitutive activation of MEK and ERK levels (Figure S17D). HUB040-N and HUB040-N-B organoids were analyzed by RNA sequencing and DNA methylation arrays. DNA methylation analysis revealed a significant increase in global hypermethylation in HUB040-N-B organoids compared to HUB040-N (Figure S18A). Specifically, 4 DMRs were identified, all located within gene bodies (Table S4), with the most significant DMR annotated to *TCF4*. In addition to *TCF4*, differentially methylated CpG sites were also identified in the gene bodies of *LGR5* and *EPHB2*, as well as the promoter region of *FAM3D* (Figure S18B). These epigenetic changes were associated with transcriptional alterations: TCF4 and FAM3D expression were increased, EPHB2 expression remained unchanged, and LGR5 expression was reduced in HUB040-N-B organoids (Figure S18C). RNA sequencing analysis revealed that the WNT signature^26^ was upregulated in HUB040-N-B organoids versus HUB040-N organoids (Figure S18C). At the protein level, western blot analysis demonstrated increased expression of key WNT target genes AXIN2 and c-MYC, while FAM3D expression was reduced in HUB040-N-B organoids (Figure S18D). Thus, the BRAF^V600E^ mutation not only maintains high WNT signaling in a late-stage cancerous background, but is also capable to promote WNT signaling in otherwise wildtype (healthy) colon organoids.

### Pharmacological DNA demethylation suppresses WNT signaling in BRAF^V600E^ organoids

**Figure 5:**
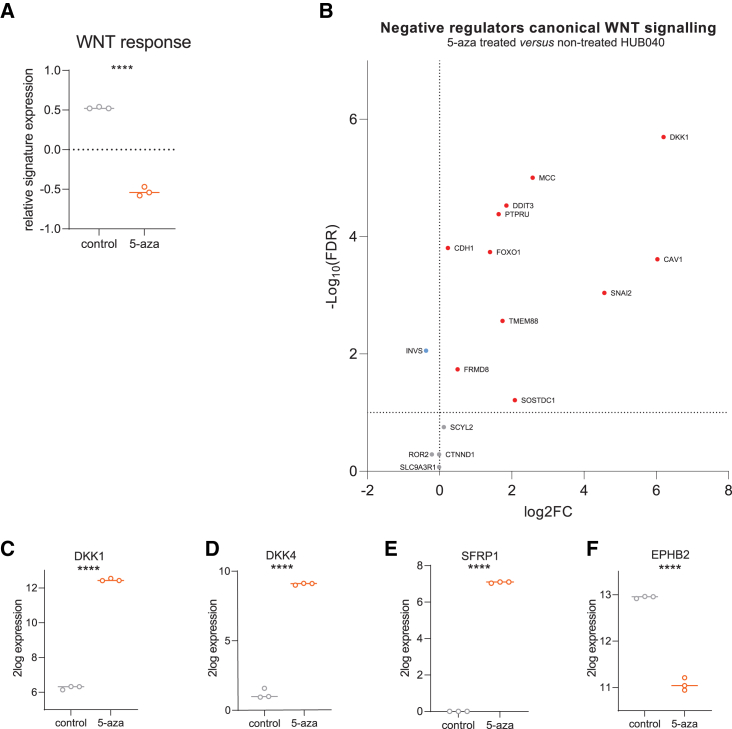
Pharmacological DNA demethylation suppresses WNT signaling in BRAF^V600E^ organoids (A) Relative signature expression (z-scores) of WNT target gene signature (Van Der Flier et al.) in 5-aza treated versus non-treated HUB040. (B) Volcano plot of mRNA expression data of downregulated negative regulators of canonical WNT signaling (found in HUB040 and NC versus BC1 and BC2) in 5-aza treated versus non-treated HUB040. (C–F) 2log expression of (C) DKK1, (D) DKK4, (E) SFRP1, and (F) EPHB2 in 5-aza treated versus non-treated HUB040. Unpaired *t*-test ∗∗∗∗*p* < 0.0001. FC, fold change; FDR, false discovery rate.

The above results demonstrate that the V600E mutation in BRAF is required for maintenance of the aberrant DNA methylation landscape (including promoters of WNT antagonist genes and gene bodies of crucial WNT target genes and key WNT-effector *TCF4*) and for maintaining high levels of WNT signaling. To assess the causal relationship between the two phenomena, the HUB040 PDO was treated with the DNA demethylating agent 5-aza-2′-deoxycytidine (5-aza). After 7 days of treatment, RNA was isolated from treated and non-treated PDOs, and treatment-induced gene expression changes were analyzed by RNA sequencing. Treatment with 5-aza severely reduced expression of the WNT target gene signature^26^ (similar as in BC1 and BC2) (Figures 5A and S19). Of the 16 WNT antagonist genes (GO_0090090) (Table 1) that were upregulated in BC1 and BC2, 11 genes were also transcriptionally upregulated following 5-aza treatment (Figure 5B), including *DKK1*, one of the most upregulated genes in 5-aza treated HUB040 organoids (Figures 5C and S20). Treatment with 5-aza also induced significant transcriptional upregulation of the WNT antagonist genes *DKK4* and *SFRP1* (Figures 5D, 5E, and S20), although these were not upregulated in BC1 and BC2.

5-aza treatment of HUB040 resulted in upregulation of 31% of genes (*p* < 0.01) with hypermethylated promotors (Figure S21A), of which half were also upregulated in V600E corrected organoids (Figure S21B). Gene body hypermethylation of *LGR5* and *EPHB2* correlated with transcriptional upregulation in HUB040 and NC versus BC1 and BC2 (Figure 4D). 5-aza treatment of HUB040 was sufficient to cause significant downregulation of EPHB2, to levels observed in BC1 and BC2 (Figure 5F). However, 5-aza did not affect LGR5 expression. Thus, pharmacological DNA demethylation is sufficient to suppress WNT signaling in BRAF^V600E^ PDOs, and thereby phenocopies correction of the BRAF^V600E^ mutation.

## Discussion

In this study, we demonstrate that the BRAF^V600E^ mutation is essential to maintain the transformed phenotype of PDOs derived from a late-stage colon tumor. Despite the continued presence of oncogenic mutations in TP53, SMAD4, and RNF43, BC1 and BC2 organoids had a severely diminished growth capacity in the absence of EGF, displayed strongly reduced WNT signaling, and had lost tumorigenic capacity. The major changes in tumor biology observed with correction of the BRAF^V600E^ mutation are reminiscent of the reversal of CRC cells to normal cells following re-expression of wildtype APC.^38^

RNF43 is a negative regulator of WNT signaling by targeting WNT receptors on the cell surface for ubiquitination-mediated degradation.^8^ Therefore, truncating (frameshift) mutations in this gene are suspected to cause dysregulation of WNT signaling in cancer.^39^^,^^40^^,^^41^ Currently, it remains unclear to what extent truncating mutations in the *RNF43* gene contribute to activation of WNT signaling in cancer. Introduction of missense mutations (which lead to loss of function) in RNF43 revealed that RNF43 functions as a potent negative feedback regulator of WNT signaling.^9^^,^^42^ However, the most prevalent mutant RNF43 protein in CRC (G659fs) retains the capacity to suppress WNT signaling,^10^^,^^43^^,^^44^ and may promote PI3K signaling instead.^45^ The data presented in this study show that the V600E mutation in BRAF, rather than the frameshift mutation in RNF43, promotes WNT signaling activity. While correction of RNF43 resulted in increased dependency on EGF, WNT, and RSPO supplementation for optimal growth, RNF43-corrected organoids remained viable when supplemented with either EGF alone or WNT/RSPO alone,^22^ suggesting that WNT/RSPO are not strictly essential for their survival. In contrast, BRAF-V600E corrected organoids, despite their ability to grow independently of WNT ligands *in vitro*, completely lost tumorigenic potential *in vivo*. BRAF-mutant organoids exhibited significantly higher WNT signaling than BRAF-corrected organoids, which showed marked downregulation of key WNT pathway genes (*LGR5*, *EPHB2*, and *TCF4*) regardless of growth conditions. Conversely, RNF43-corrected clones maintained a stable WNT response signature, indicating that RNF43 primarily regulates WNT ligand dependency rather than directly enhancing WNT signaling. These findings underscore the cooperative role of BRAF^V600E^ and RNF43 mutations in driving a fully transformed, metastatic phenotype, with BRAF^V600E^ serving as a potent enhancer of WNT signaling in colon cancer.

The V600E mutation in BRAF induces genome-wide DNA methylation changes that contribute to oncogenic transformation.^13^^,^^46^ Negative WNT regulators are often subject to promoter hypermethylation in CRC.^28^ Our results indicate that BRAF (V600E)-induced methylation changes underlie activation of WNT signaling in BRAF/RNF43 mutant tumors, and that this involves three modes of methylation-controlled gene expression: First, BRAF (V600E) induces mechanisms that result in the hypermethylation of the promoters of multiple negative regulators of WNT signaling (including *FAM3D* and *CTNND1*), thereby causing gene silencing. FAM3D expression is significantly higher in normal colon tissues compared to CRC tissues, with the lowest levels observed in stage IV CRC.^29^ Previous research has demonstrated that FAM3D deficiency (*Fam3D*^−/−^ mice) led to elevated β-catenin levels.^29^ Our RNA-seq data identified *FAM3D* as the top differentially expressed gene (*BRAF*-M < *BRAF*-corrected), and our methylation data indicated that its promoter is the most hypermethylated (*BRAF*-M > *BRAF*-corrected). This suggests that FAM3D likely plays a pivotal role in BRAF-V600E mutant CRCs. Additionally, its downregulation in larger datasets supports this notion. Given the association of FAM3D deficiency with increased WNT activation, which parallels our observations in BRAF^V600E^-mutant organoids, it is highly probable that FAM3D exerts similar effects in BRAF-V600E mutant CRCs. While mechanistic studies on how FAM3D functions are lacking, it is noteworthy that FAM3D deletion can induce WNT pathway activation. Second, BRAF (V600E) induces mechanisms that result in the hypermethylation of the gene bodies of WNT target genes (including *LGR5* and *EPHB2*), which is associated with stimulation of gene expression.^15^^,^^16^^,^^17^^,^^18^^,^^19^^,^^20^ Indeed, 25% of the genes with hypermethylated gene bodies in BRAF^V600E^ organoids was transcriptionally induced and 37% of the genes with hypermethylated promoters was transcriptionally silenced. LGR5 enhances WNT signaling in the presence of the ligand *R*-spondin by removing two E3 ligases (RNF43 and ZNRF3). LGR5 overexpression, as a result of gene body hypermethylation, could potentiate the removal of these negative WNT regulators. In this context, the removal of ZNRF3 by LGR5 could potentiate WNT signaling in BRAF/RNF43 mutant CRCs. Third, in both BRAF^V600E^ and HUB040-N-B organoids gene body methylation of *TCF4* was observed. TCF4 is essential for activation of canonical WNT signaling,^47^^,^^48^ as canonical WNT-target gene expression is initiated by formation of the b-catenin·TCF4/LEF-1 complex. Increased expression of TCF4 suggests that more TCF4 molecules are available in the nucleus, potentially allowing for greater binding to WNT-responsive elements (WREs) on DNA. This could provide more opportunities for β-catenin to form complexes with TCF4, thereby enhancing the activation of WNT target genes and contribute to activating WNT signaling in BRAF-V600E mutant CRC. Given lower b-catenin levels in serrated pathway CRCs compared to APC-mutant carcinomas, TCF4 overexpression could efficiently drive WNT activation in a manner that is favorable to these tumors. The fact that this phenomenon was observed with both CRISPR strategies (i.e., correction of the V600E mutation in colon cancer organoids and knock-in of the V600E mutation of healthy colon organoids from the same patient), suggests a key role for BRAF^V600E^ in gene body hypermethylation of *TCF4*, leading to activation of WNT signaling in colon cancer.

Introduction of the BRAF^V600E^ mutation in healthy colon organoids revealed gene body hypermethylation in *TCF4*, as well as differential methylation in other WNT pathway related genes, including *LGR5*, *EPHB2*, and *FAM3D*. These results highlight a potential role for the BRAF-V600E mutation in modifying the methylation landscape of genes involved in WNT signaling, leading to altered expression of WNT target genes. The differential methylation observed in TCF4 and FAM3D appears to drive changes in their expression, whereas for LGR5 and EPHB2, the lack of corresponding changes in expression suggests additional layers of regulation are at play. While it is clear that BRAF^V600E^ influences DNA methylation and WNT signaling pathways, assessing the relative contribution of each specific methylation event to altered WNT target gene expression remains a complex challenge. In addition, it is important to recognize that the mere introduction of an oncogene is often insufficient to induce a fully cancerous phenotype in these organoids.^49^^,^^50^^,^^51^ Cancer development typically involves the accumulation of multiple genetic and epigenetic alterations. It is conceivable that the presence of additional mutations, such as those affecting TP53 or SMAD4, might provoke a more pronounced cancerous phenotype and lead to greater epigenetic aberrations. Furthermore, aging-related epigenetic changes could serve as another prerequisite for oncogenic transformation,^32^ potentially facilitating or amplifying the epigenetic effects of the BRAF-V600E mutation. Nonetheless, the significant gene body methylation observed in the WNT effector gene *TCF4* following the introduction of BRAF-V600E mutation underscores the potential of this mutation to impact WNT signaling activation. This suggests that while the BRAF-V600E mutation alone can initiate certain epigenetic changes, a fully transformed phenotype likely requires a combination of multiple oncogenic events. Furthermore, treatment of BRAF^V600E^ mutant CRC organoids with encorafenib showed no alterations in WNT signaling. Although both correction of the V600E mutation and encorafenib treatment affect the MAPK pathway, our observations indicate that the impact of encorafenib on WNT signaling might not be apparent in the short term. One possible explanation for these findings is the differential activation of feedback mechanisms following mutation correction and BRAF inhibition. Upon BRAF inhibition, the downstream MAPK pathway is initially suppressed, which could activate compensatory signaling pathways or feedback loops to restore cellular balance. These feedback mechanisms might include upregulation of alternative signaling pathways that could counteract the effects of MAPK pathway inhibition on WNT signaling. Additionally, WNT pathway regulation might be influenced by long-term adaptive (indirect) responses following mutation correction that are not immediately evident in short-term experiments using BRAF inhibitors.

The observed changes in DNA methylation within the promoters of WNT antagonist genes and the gene bodies of WNT effector/target genes suggest a mechanism linked to CIMP. Although the exact mechanism remains to be fully elucidated, it is possible that the constitutive activation of the MAPK pathway, driven by the BRAF-V600E mutation, leads to specific epigenetic modifications. This could occur through the upregulation of transcription factors and other regulatory proteins that directly interact with the DNA methylation machinery, resulting in the hypermethylation of specific CpG islands. It is intriguing that mutant BRAF is closely associated with DNA methylation alterations, whereas KRAS mutations, despite affecting the same signaling pathway, show this association to a much lesser extent.

Despite the downregulation of WNT signaling upon correction of the V600E mutation, V600E-corrected organoids exhibited continued independence from exogenous WNT ligands. This independence could potentially stem from the intrinsic capacity of cancerous organoids to produce WNT ligands autonomously. Prior findings^22^ indicating the high sensitivity of HUB040 organoids to porcupine inhibition further support the notion that these organoids possess the ability to self-secrete this ligand. Moreover, the introduction of the V600E mutation into healthy colon organoids did not confer independence from exogenous WNT ligands, suggesting that in this case additional cancerous mutations may be required to establish a self-sufficient microenvironment conducive to tumor cell proliferation. These findings collectively suggest that BRAF^V600E^ augments WNT signaling, presumably through altering the epigenetic landscape, but also that other factors, including mutant RNF43, may confer WNT ligand independence.

The current standard-of-care treatment for metastatic BRAF-V600E mutant CRCs is a combination of the BRAF inhibitor encorafenib with the epidermal growth factor receptor (EGFR) inhibitor cetuximab.^52^^,^^53^ Although this treatment was superior compared to standard chemotherapy, resistance is commonly observed and the median overall survival of patients with BRAF-V600E mutant CRC remains very poor (∼9 months).^52^ The identification of DNA methylation as the central mechanism of oncogenic transformation by BRAF (V600E) opens new possibilities for developing alternative therapeutic strategies.

Demethylation treatment of BRAF^V600E^ organoids with 5-aza caused a strong decrease in WNT signaling and a complete loss of their regenerative capacity (see accompanying paper^54^). Most of the WNT antagonist genes that were downregulated in BRAF^V600E^ organoids were transcriptionally induced (de-repressed) following 5-aza treatment. Interestingly, while we did not detect hypermethylation of the *DKK1* promoter in our initial analysis, its expression was still induced by 5-aza treatment. This suggests that there is some degree of DNA methylation associated with the *DKK1* gene, even if it was not sufficient to be detected as a DMR initially. This finding aligns with previous studies showing *DKK1* promoter hypermethylation in CRC.^55^ While DKK1 is downregulated in BRAF-mutant organoids and in BRAF-mutant CRCs in larger datasets (such as the CRC TCGA dataset^30^ and the colon cancer dataset from Marisa et al.^31^), this does not necessarily imply that BRAF^V600E^ directly causes this downregulation. Additionally, not all BRAF^V600E^-induced methylation changes were reversible by 5-aza treatment. For example, 5-aza caused suppression of EPHB2 expression following 5-aza treatment, as was observed following *BRAF*^V600E^ correction, but had no effect on LGR5 expression. In addition, WNT antagonist genes *DKK4*, and *SFRP1* were upregulated following 5-aza treatment in *BRAF*^V600E^ PDO HUB040, but expression of these genes was unaffected by correction of the V600E mutation in the same PDO. One of the possible explanations could be that when the mutation is corrected, some epigenetic changes that were established during the presence of the mutation may be sustained, driven by other (currently unknown) factors. Nevertheless, the considerable overlap in phenotypic and molecular changes induced by correction of the V600E mutation and 5-aza treatment forms the basis for developing epigenetic therapies for BRAF-V600E mutant CRC. DNA demethylating drugs (azacitidine and decitabine) are currently FDA-approved for the treatment of patients with myelodysplastic syndrome (MDS) and acute myeloid leukemia (AML).^56^ The clinical response of demethylating drugs in solid tumors can be beneficial for individual patients, but the overall benefit is limited, possibly because most studies fail to employ patient selection^57^ or because the drugs fail to induce demethylation of tumor DNA.^58^ We propose that the BRAF^V600E^ mutation in CRC may be associated with response to demethylating agents. In the accompanying report, we provide evidence that treatment with DNA demethylating drugs leads to a complete loss of regenerative capacity of BRAF^V600E^ PDOs.^54^ Clinical studies in which epigenetic therapies are tested in pre-selected patients with BRAF^V600E^ mutated tumors are now warranted.

### Limitations of the study

This study explored the role of the BRAF^V600E^ mutation in activation of WNT signaling within CRC. While our study provides valuable insights into the role of the BRAF^V600E^ mutation in driving WNT pathway activation and tumorigenesis in CRC, there are several limitations that should be acknowledged. First, our findings are based on experiments conducted using PDOs derived from a single late-stage colon tumor. The genetic and molecular landscape of BRAF-V600E mutant CRC can vary significantly between patients, and our results may not fully represent the heterogeneity observed in clinical populations. However, most of the findings related to WNT signaling activation were verified in larger CRC datasets where BRAF-mutant and BRAF-wildtype CRCs were compared. Second, while our study suggests potential therapeutic strategies targeting DNA methylation to suppress WNT pathway activation in BRAF-V600E mutant CRC, further preclinical work in PDOs and mouse models is needed to confirm the generic efficacy of such approaches in BRAF-V600E mutant CRC. Third, our findings reveal that BRAF^V600E^ augments WNT signaling activation by indirectly inducing changes in widespread DNA methylation without elucidating the exact underlying mechanisms. Future studies should elucidate these mechanisms, which may pave the way for novel strategies to combat this aggressive subtype of CRC.

## Resource availability

### Lead contact

Further information and requests for resources and reagents should be directed to and will be fulfilled by the lead contact, Onno Kranenburg (o.kranenburg@umcutrecht.nl).

### Materials availability

Plasmids generated in this study are available from the lead contact. PDOs may be obtained from the lead contact with a completed materials transfer agreement.

### Data and code availability

• The Infinium MethylationEPIC BeadChip data generated in this study have been deposited in ArrayExpress, accessions E-MTAB-13283 (BRAF-V600E correction) and E-MTAB-13290 (BRAF-V600E knock-in). The RNA-seq data generated in this study have been deposited in ArrayExpress, accessions E-MTAB-13806 (BRAF-V600E correction), E-MTAB-14298 (BRAF-V600E correction with advanced starvation) and E-MTAB-13842 (BRAF-V600E knock-in). RNA sequencing data are also accessible under request in the “R2: Genomics Analysis and Visualization Platform (http://r2.amc.nl)”.
• This paper does not report original code.
• Any additional information required to reanalyze the data reported in this work paper is available from the lead contact upon request.

## Acknowledgments

The authors thank the Pathology department at the UMC Utrecht for running the Infinium MethylationEPIC array and ddPCR for the BRAF-V600E mutation. We acknowledge the Utrecht Sequencing Facility (USEQ) for providing RNA sequencing service and data. This work was supported by the 10.13039/501100004344Dutch Foundation for medical scientific research ZonMW TOP Grant 91218050 (to M.M.M., O.K., and H.J.G.S.). M.M.M is funded by 10.13039/501100021821Oncode Institute, which is partly financed by the 10.13039/501100004622Dutch Cancer Society (KWF), KWF Grant 13112, TKI/10.13039/501100004622KWF grant 14853 and the NWO Gravitation project IMAGINE!

## Author contributions

L.E.B., J.M.B., H.J.G.S., M.M.M., and O.K. conceptualized the project. L.E.B. and O.K. designed and analyzed experiments. J.M.B., E.K., A.V., J.B.P., and N.F. provided methodological expertise. I.H.M.B.R., H.J.G.S., M.M.M., and O.K. provided supervision for this study. L.E.B. and O.K. wrote the manuscript. All authors reviewed and edited the final version of the manuscript.

## Declaration of interests

The authors declare no competing interests.

## STAR★Methods

### Key resources table

**Table:** 

REAGENT or RESOURCE	SOURCE	IDENTIFIER
**Antibodies**		

Phospho-MEK1/2 (Ser217/221)	Cell Signaling Technology	9121; RRID:AB_331648
MEK1/2	Cell Signaling Technology	9122; RRID:AB_823567
Phospho-p44/42 MAPK (Erk1/2) (Thr202/Tyr204)	Cell Signaling Technology	9101; RRID:AB_331646
p44/42 MAPK (Erk1/2) (137F5)	Cell Signaling Technology	4695; RRID:AB_390779
FAM3D	R&D Systems	AF2869; RRID:AB_2101496
β-actin	Novus Biologicals	NB600-501; RRID:AB_343280
goat anti-mouse HRP	Dako	P0447; RRID:AB_2617137
goat anti-rabbit HRP	Dako	P0448; RRID:AB_2617138
anti-goat HRP-conjugated	R&D Systems	HAF109; RRID:AB_357236

**Biological samples**		

Patient-Derived Organoids (PDOs) – CRC and healthy colon organoids	Hubrecht Organoid Technology	HUB-Cancer TcBio#12–09

**Chemicals, peptides, and recombinant proteins**		

Dispase II	Roche	12273600
TrypLE™ Express Enzyme	ThermoFisher Scientific	12604013
Matrigel	Corning	354234
Advanced DMEM/F12	ThermoFisher Scientific	12634010
B27 supplement	ThermoFisher Scientific	17504044
N-Acetyl-L-Cysteine	Sigma-Aldrich	A9165
Nicotinamide	Sigma-Aldrich	N0636
A83-10	Tocris	2939
SB202190	Sigma-Aldrich	S7067
EGF (Recombinant Human EGF)	PeproTech	AF-100-15
Glutamax	ThermoFisher Scientific	35050-038
HEPES	ThermoFisher Scientific	15630-056
Penicillin/Streptomycin	Lonza	DE17-602E
Noggin conditioned medium	Prepared in house	N/A
R-spondin1 conditioned medium	Prepared in house	N/A
WNT conditioned medium	Prepared in house	N/A
ROCK inhibitor (Y-26732)	Sigma-Aldrich	129830-38-2
Decitabine (5-Aza-2′-deoxycytidine)	AbMole	M2052
Protease inhibitor cocktail	Cell Signaling Technology	5871

**Critical commercial assays**		

QIAamp DNA Micro Kit	Qiagen	56304
ddPCR BRAF V600 Screening Kit	Bio-Rad	12001037
CellTiter-Glo® 3D Cell Viability Assay	Promega	G9681
RNeasy® Mini Kit	Qiagen	74004
Infinium Human MethylationEPIC Beadchip	Illumina	20087706

**Deposited data**		

MethylationEPIC data - BRAF-V600E correction	ArrayExpress	E-MTAB-13283
MethylationEPIC data - BRAF-V600E knock-in	ArrayExpress	E-MTAB-13290
RNAseq data - BRAF-V600E correction	ArrayExpress	E-MTAB-13806
RNAseq data - BRAF-V600E correction (adv starvation)	ArrayExpress	E-MTAB-14298
RNAseq data - BRAF-V600E knock-in	ArrayExpress	E-MTAB-13842

**Experimental models: Organisms/strains**		

*NOD*.*Cg-Prkdc*^*scid*^*Il2rg*^*tm1Wjl*^*/SzJ* (NSG) mice	Charles River	n/a

**Oligonucleotides**		

gRNA-BRAF-V600E correction-hLbCpf1 (DR) sequence (5′-AATTTCTACTAAGTGTAGAT-3′) guide sequence (5′-GTCTAGCTACAGAGAAATCTCGA-3′)	This paper	N/A
Pair1_BRAF-exon 15_forward 5′-GGAGAGCAGGATACCACAGC-3′	This paper	N/A
Pair1_BRAF-exon 15_reverse 5′-CCACCGGTAGGCGCCAAC-3′	This paper	N/A
Pair2_BRAF-exon 15_forward 5′-CCTCTGACCTTGCTCAGTGG-3′	This paper	N/A
Pair2_BRAF-exon 15_reverse 5′-TGGATCCAGACAACTGTTCAA-3′	This paper	N/A
gRNA-BRAF-V600E KI-hCas9 5′-GAAGACCTCACAGTAAAAAT-3′	This paper	N/A

**Recombinant DNA**		

phU6-gRNA	Addgene	53188
pcDNA3.1-hLbCpf1	Addgene	69988
BRAF-V600E_correction template	This paper	N/A
hCas9	Addgene	41815

**Software and algorithms**		

shinyÉPICo	https://bioconductor.org	N/A
	Morante-Palacios et al. https://doi.org/10.1093/bioinformatics/btaa1095	
R (version 4.2.1)	https://cran.r-project.org	N/A
GraphPad Prism 9	GraphPad software	N/A
R2 Genomics Analysis and Visualization Platform	R2; http://r2.amc.nl	N/A

### Experimental model and study participant details

#### CRC/healthy colon patient-derived organoid cultures

The collection and processing of human colorectal cancer and normal colon tissues (HUB-Cancer TCBio protocol ID number 12-093) was approved by the Biobank Research Ethics Committee of the University Medical Center Utrecht (Utrecht, The Netherlands). Written informed consent from the donors for research use of tissue in this study was obtained prior to the acquisition of the specimen according to the principles expressed in the Declaration of Helsinki.

CRC/healthy colon patient-derived organoids with identifiers HUB-02-B2-040 (CRC) and HUB-02-A2-040 (healthy colon) were previously established and characterized.^59^ The organoids utilized in this study originated from a stage IV female colon cancer patient with peritoneal metastases. The tumour was classified as microsatellite stable (MSS). Human colon (cancer) organoids were cultured as described previously.^60^ The organoids were embedded in Matrigel (Corning) and cultured with advanced DMEM/F12 medium (Gibco) with 1x Penicillin/Streptomycin (Lonza), 10 mM HEPES (Invitrogen) and 2 mM Glutamax (Gibco). The basal medium was further supplemented with 1% Noggin conditioned medium, 1x B27 Supplement (Gibco), 10 mM Nicotinamide (Sigma-Aldrich), 1.25 mM N-acetylcysteine (Sigma-Aldrich), 500 nM A83-01 (Tocris), and 10 μM SB202190 (Sigma-Aldrich). After organoid transfection the growth medium of *BRAF*^V600E^ and *BRAF*^E600V^ organoids was supplemented with 50 ng/mL EGF (PreproTech). HUB040-N and HUB040-N-B organoids were cultured with the extra supplementation of 1% surrogate WNT conditioned medium, 20% Rspo1 conditioned medium, and 50 ng/mL EGF (PreproTech). Organoids were split every week through TrypLE Express (Gibco) treatment. Culture medium after splitting was supplemented with 10 μM Y-27632 dihydrochloride (Sigma-Aldrich). For selection of *BRAF*^E600V^ and HUB040-N-B organoids 1 μg/μL puromycin was supplemented in the growth medium.

#### Animals

Animal experiments were approved by the Competent Authority, The Netherlands (License number AVD115002016614), which is advised by the Animal Ethics Committee. Animal work protocol (614-01-22) was approved by the Animal Welfare Body and was performed in accordance with the Dutch Law on Animal Experiments and the European Directive 2010/63/EU. All *in vivo* experiments were performed in healthy 8–10 week old 25–30 g male *NOD*.*Cg-Prkdc*^*scid*^ *Il2rg*^*tm1Wjl*^*/SzJ* (NSG) mice, supplied by Charles River. Animals were randomly allocated in groups of 5 mice into individually ventilated cages. HUB040/NC/BC1/BC2 organoids (4 experimental groups) were dissociated 5 days after passaging into single cells by TrypLE Express. A cell-matrigel (1:1) suspension was prepared with 5 × 10^6^ cells/ml. Using a 1 mL syringe and 25G 0.5 × 16 mm needles, 100 μL of the suspension were subcutaneously injected into the right flank of animals (5 animals per organoid line, 20 animals in total). Animals were sacrificed when the tumour volume reached 1.5 mm3, as measured by V = 1/2 × (smaller diameter^2^ × larger diameter). Animal welfare was monitored by physical appearance, behaviour, and body weight.

### Method details

#### Gene engineering of organoids and genotyping

To correct the BRAF^V600E^ mutation in the HUB040 CRC PDO, an estimated 1 × 10^6^ cells of this PDO were electroporated with the single-guide RNA plasmid, pcDNA3.1-hLbCpf1 (Addgene, 69988) and a BRAF-V600E correction template plasmid. To specifically target the mutant allele, a guide RNA was designed to solely bind the mutant sequence.^61^ The gRNA for hLbCpf1 is composed of the 20-nt direct repeat (DR) sequence (5′-AATTTCTACTAAGTGTAGAT-3′) followed by a 23-nt guide sequence (5′-GTCTAGCTACAGAGAAATCTCGA-3′). Oligonucleotide duplexes corresponding to spacer sequences were annealed and ligated into BbsI-digested phU6-gRNA (Addgene, 53188). For the generation of the BRAF-V600E correction template site-directed mutagenesis was performed on a BRAF-V600E mutation template that was generated earlier.^62^ In short, for the generation of the donor template, genomic DNA from P18T organoids^60^ was used to PCR amplify the *BRAF* homology arms using high-fidelity Phusion Polymerase (New England Biolabs). The 5′ homology arm spans the region Chr7:140753392-140753973, the 3′ homology arm spans the region Chr7:140752465-140753206. Gene block fragments (idtDNA) were used to generate 5′ homology arms containing silent mutations. The gene block fragments and homology arms were cloned into a pBlueScript plasmid expressing a 3229-bp AATPB:PGKpuroDtk selection cassette.^63^ Clonal lines were established under puromycin selection, in which single cells were grown into single organoid structures that were individually picked and expanded. Genomic DNA was extracted from expanded single organoids using the QIAamp DNA Micro Kit (Qiagen) and correction of the BRAF^V600E^ mutation was confirmed by Sanger sequencing and the ddPCR BRAF V600 Screening Kit (Bio-Rad). The sequences of primers used for Sanger sequencing are: Pair 1 (5′-3′) Fw: GGAGAGCAGGATACCACAGC and Rv: CCACCGGTAGGCGCCAAC, and Pair 2 (5′-3′) Fw: CCTCTGACCTTGCTCAGTGG and Rv: TGGATCCAGACAACTGTTCAA.

Correction of the RNF43-P441fs mutation in HUB040 was performed as previously described.^22^

For generation of HUB040-N-B organoids an estimated 1 × 10^6^ cells of HUB040-N organoids were electroporated with the single-guide RNA plasmid (5′-GAAGACCTCACAGTAAAAAT-3′) ligated into BbsI-digested phU6-gRNA (Addgene, 53188), a human codon-optimized Cas9 expression plasmid (Addgene, 41815), and the BRAF-V600E mutation template.^62^ Clonal lines were established under puromycin selection and introduction of the BRAF^V600E^ mutation was confirmed by Sanger sequencing using Primer pair 1 and 2.

#### EGF dependency assays

##### Organoid growth assessment

HUB040, NC, BC1, and BC2 organoids were dissociated into single cells with TrypLE Express treatment for 3 min at 37°C. Single cells were pelleted (1800 RPM, 3 min), embedded in Matrigel and culture medium was added, supplemented with or without 50 ng/mL EGF. Culture medium was refreshed every 3 days and organoids were kept in culture for 9 days.

##### EGF stimulation

7-day-old HUB040, NC, BC1, and BC2 organoids were starved overnight (16 h) from essential medium supplements and kept in basal medium. After overnight starvation, organoids were collected from Matrigel by enzymatic digestion using 1 mg/mL dispase (Gibco) for 10 min at 37°C, were pelleted, and resuspended in 1 mL basal medium supplemented with or without 50 ng/mL EGF. After EGF stimulation, organoids were washed with PBS and pelleted two times.

##### EGF rescue experiment

HUB040, NC, BC1, and BC2 organoids were dissociated into single cells with TrypLE Express (Gibco) treatment for 3 min at 37°C. Single cells were pelleted, embedded in Matrigel (Corning) and culture medium supplemented with 50 ng/mL EGF was added. Organoids were grown for 5 days with the supplementation of EGF, after which organoids were starved for 7 consecutive days from EGF. After EGF starvation, organoids were cultured in culture medium supplemented with EGF for 6 days (EGF rescued) or starved for another 6 days from EGF (no rescue). At the end of the experiment viability of organoids was measured with CellTiter-Glo 3D assays (Promega) and luminescence levels were measured using a SpectraMax M5 microplate reader (Molecular Devices).

#### Western Blot

Cell lysates were prepared with RIPA lysis buffer in presence of protease inhibitor cocktail (CST) and 1 mM phenylmethylsulfonyl fluoride, incubated for 30 min on ice, and centrifuged at 12,000 rpm at 4°C for 10 min to pellet cell debris. Protein concentration was measured using a BCA kit (Thermo Fisher) and 10 or 20 μg of total protein were run on a 10% or 15% SDS-PAGE gel at 120 V until loading dye reached the bottom of the gel. The proteins were then transferred to a nitrocellulose membrane (Bio-Rad) using the BioRad Transblot Turbo Transfer System (BioRad, 1704155) and blocked with 5% BSA diluted in TBS-T (50 mM Tris-HCl/150 mM NaCl (pH 7.6)/0.1% Tween 20) for 1 h at room temperature. Membranes were rocked overnight at 4°C with primary antibodies diluted 1:1000 in blocking solution. Membranes were probed with antibodies directed against phospho-MEK1/2 (CST, 9121, 1:1000), MEK1/2 (CST, 9122, 1:1000), phospho-ERK1/2 (CST, 9101, 1:1000), ERK1/2 (CST, 4695, 1:1000), and FAM3D (R&D Systems, AF2869, 1:1000). GAPDH (CST, 5174, 1:1000) and β-actin (Novus Biologicals, NB600-501, 1:20000) were used as an internal control. Primary antibodies were revealed using goat anti-mouse HRP (Dako, P0447, 1:2000), goat anti-rabbit HRP (Dako, P0448, 1:1000) or anti-goat HRP-conjugated (R&D Systems, HAF109, 1:1000) and detected with WesternBright ECL (Advansta).

#### RNA sequencing and data Analysis

Total RNA was extracted from BRAF^V600E^ (HUB040 and NC) and BRAF^E600V^ (BC1 and BC2) organoids, HUB040-N and HUB040-N-B organoids, and HUB040 organoids treated with decitabine (4 μM), and untreated (DMSO control).

Organoids were collected from Matrigel by enzymatic digestion using 1 mg/mL dispase (Gibco) for 10 min at 37°C and were lysed in RLT-buffer (Qiagen) supplemented with 1% β-mercaptoethanol. Total RNA was isolated using the RNeasy Mini Kit (Qiagen) according to the manufacturer’s protocol. DNase (Qiagen) treatment was included to avoid possible genomic DNA contamination. Extracted RNA was quantified using a Qubit fluorometer and quality assessed using bioanalyzer quality control. Libraries were prepared using Truseq RNA stranded polyA (Illumina) according to the manufacturer’s directions. Sequencing was performed on a NextSeq2000 (Illumina; 1 × 50 bp) and base calling performed using RTA (Illumina). Quality control of raw reads was done using FastQC.

The RNAseq datasets were uploaded into R2 (http://r2.amc.nl) for subsequent bioinformatics analyses and are available on the platform. Differential gene expression analysis was performed with the DESeq2 package^64^ within the R2 environment, using a cut-off of *p* < 0.01 (ANOVA) with multiple testing correction by false discovery rate. Gene enrichment analysis was performed using ShinyGO.^65^

#### Infinium MethylationEPIC BeadChip and data analysis

Genomic DNA from BRAF^V600E^ (HUB040 and NC) and BRAF^E600V^ (BC1 and BC2) organoids, and HUB040-N and HUB040-N-B organoids was isolated using the QIAamp DNA Micro Kit (Qiagen). Bisulfite-converted DNA was amplified, fragmented, and hybridized to Illumina Infinium Human MethylationEPIC Beadchip using standard Illumina protocol. MethylationEPIC analysis was performed using the shinyÉPICo application.^66^ Using the *minfi*^67^ package within the application, array normalization was performed against parameters provided by Illumina, and non-CpG probes and probes with SNPs were excluded. Differential CpG methylation analysis was performed as described previously.^54^ β-values ranged from 0 (not methylated) to 1 (fully methylated). DMR calculation was performed with the *mCSEA* package (minProbes = 5),^68^ and an FDR cut-off of 0.01 is used for significance. The *mCSEA* package implements a method based on the Gene Set Enrichment Analysis (GSEA) to identify subtle but consistent DMRs in complex phenotypes. The main step of *mCSEA* consists in ranking all the CpG probes by differential methylation and subsequently evaluates the enrichment of CpG sites belonging to the same region in the top positions of the ranked list by applying the GSEA implementation of the fgsea package. Regions whose CpG sites are over-represented in the top or bottom of the list can be detected as differentially methylated.

#### DNA demethylation treatment of *BRAF*^V600E^ Organoids

HUB040 organoids were exposed to 4 μM decitabine (5-Aza-2′-deoxycytidine; AbMole) in growth medium for 7 days, after which RNA was extracted from treated and untreated (DMSO control) organoids.

### Quantification and statistical analysis

Statistical analysis was performed using GraphPad Prism version 9. Significances were determined using unpaired 2-tailed Student t test, unless otherwise indicated. Values are presented as means ± SD. Differences were taken to be significant at *p* < 0.05. Details about statistical analyses can be found in figure legends and in the STAR Methods.

## Supplemental information

Document S1. Figures S1–S21

Table S1. Differentially expressed genes in HUB040 and NC versus BC1 and BC2 organoids

Table S2. DMRs (promoters) in HUB040 and NC versus BC1 and BC2 organoids

Table S3. DMRs (gene bodies) in HUB040 and NC versus BC1 and BC2 organoids

Table S4. DMRs (gene bodies) in HUB040-N-B versus HUB040-N organoids
