# Supplementary material for: BRAFV600E augments WNT signaling in colorectal cancer via aberrant DNA methylation
Source: iScience. 2025 May 20;28(7):112708. doi: 10.1016/j.isci.2025.112708 (PMC12272824; doi:10.1016/j.isci.2025.112708)
Supplement: Document S1. Figures S1–S21 [file mmc1.pdf]

iScience, Volume *28*

Supplemental information

BRAF^{V600E} augments WNT signaling

in colorectal cancer via aberrant DNA methylation

Layla El Bouazzaoui, Jeroen M. Bugter, Emre Küçükköse, André Verheem, Jasmin B. Post, Nicola Fenderico, Inne H.M. Borel Rinkes, Hugo J.G. Snippert, Madelon M. Maurice, and Onno Kranenburg

Supplementary figures

A

Gene	Mutation
ARID1A	Q473*
BRAF	V600E
SMAD4	F339L, R416S
TP53	S215I
*RNF43**	P441A*fs**63
FAT4	G3894V
AFDN	V682A
HERPUD1	L41=
PRDM1	G500R

B

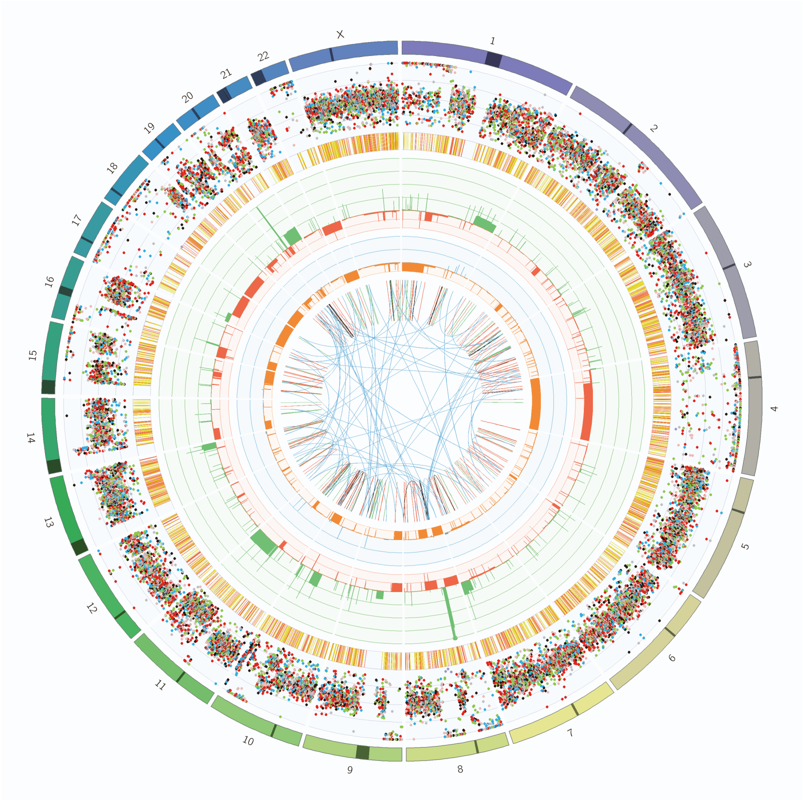

Figure S1: Genomic characterization of PDO HUB040. (A) Pathogenic cancer gene census mutations in PDO HUB040. *This specific *RNF43* mutation is not a confirmed pathogenic mutation. (B) Circos plot of PDO HUB040. The outer first circle shows the chromosomes, with darker shaded areas representing large gaps in the human reference genome. The second circle shows all tumour specific variants (incl. exon, intron and intergenic regions) and are divided into an outer ring of single nucleotide polymorphism (SNP) allele frequencies and an inner ring of short insertion/deletion (INDEL) locations. Variant allele frequencies have been corrected for tumour purity and scale from 0 to 100%. Each dot represents a single variant and are colored according to the type of base change (e.g. C>T/G>A in red) and are in concordance with the colouring used in Alexandrov et al. 2013 Nature paper that describes the use of mutational signatures. INDELs are colored yellow and red for insertions and deletions respectively. The third circle shows all observed tumour purity adjusted copy number changes, including both focal and chromosomal events. Copy number losses are indicated in red, green shows regions of copy number gain. The scale ranges from 0 (complete loss) to 6 (high level gains). If the absolute copy number is > 6 it is shown as 6 with a green dot on the diagram. The fourth circle represents the observed 'minor allele copy numbers' across the chromosome. The range of the chart is from 0 to 3. The expected normal minor allele copy number is 1, and anything below 1 is shown as a loss and represents a LOH event (orange). Minor allele copy numbers above 1 indicate amplification events of both A and B alleles at the indicated locations (blue). The innermost circle displays the observed structural variants within or between the chromosomes. Translocations are indicated in blue, deletions in red, insertions in yellow, tandem duplications in green and inversions in black.

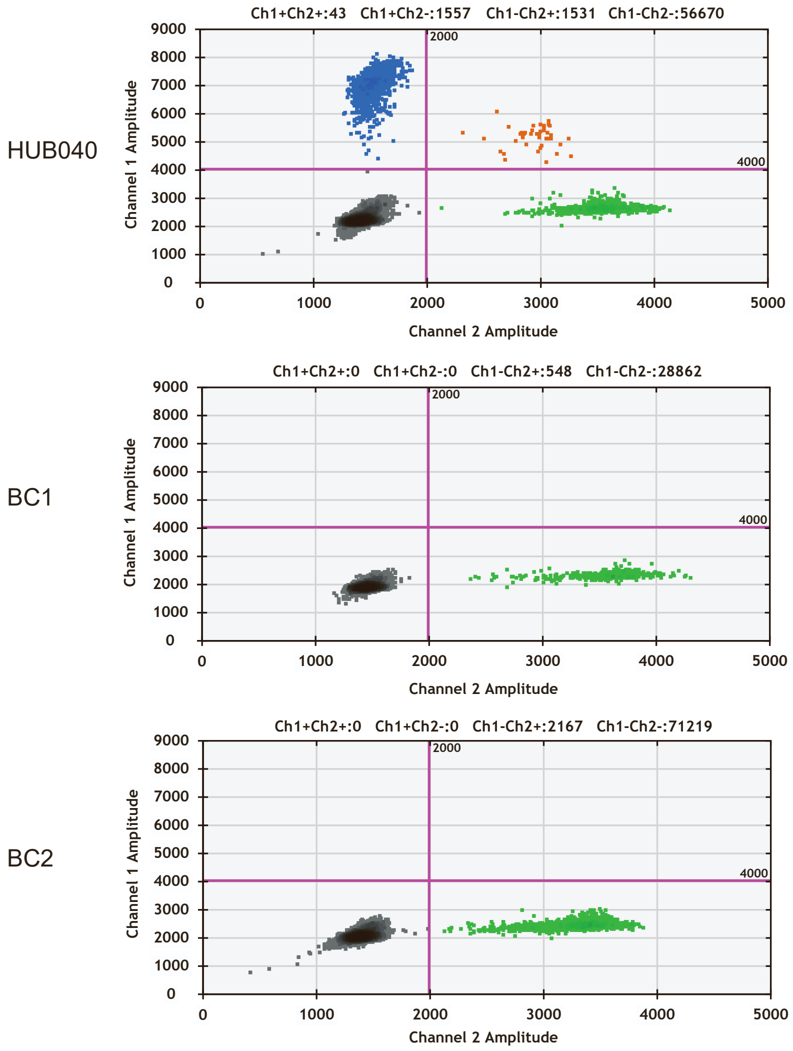

Figure S2: Droplet digital PCR results. Graphical results of ddPCR analyses of *BRAF*-V600E in HUB040 (PDO) and HUB040-derived BRAF^{E600V} organoids BC1 and BC2. Gray events represent double negative droplets, in which there was no amplification. Green events represent droplets that only contain wildtype *BRAF*. The blue cluster represents droplets containing only mutant *BRAF*, and the orange cluster represents droplets containing both wildtype and mutant *BRAF* DNA.

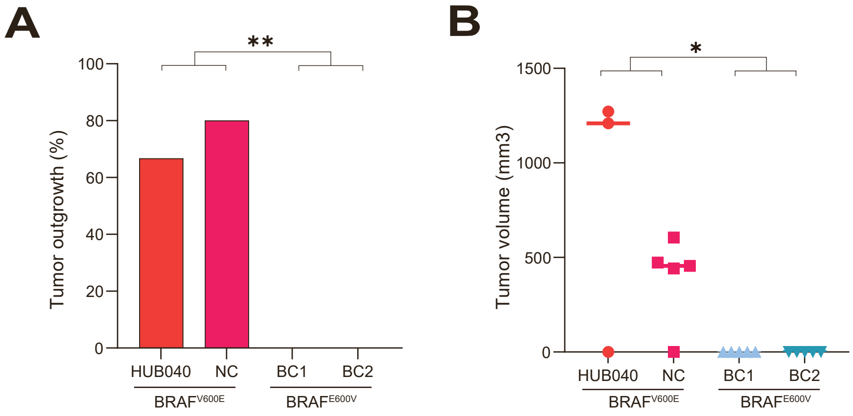

Figure S3: The V600E mutation in BRAF is indispensable for tumour-initiating capacity. Tumour growth in immunodeficient mice subcutaneously injected with $BRAF^{V600E}$ organoids (HUB040, n=3; NC, n=5) and $BRAF^{E600V}$ organoids (BC1, n=5; BC2, n=5). (A) Tumour outgrowth with injected HUB040 and NC, but no outgrowth with BC1 and BC2. (B) Final volumes of subcutaneous tumours were measured using callipers. Unpaired *t*-test *P < 0.05, **P < 0.01.

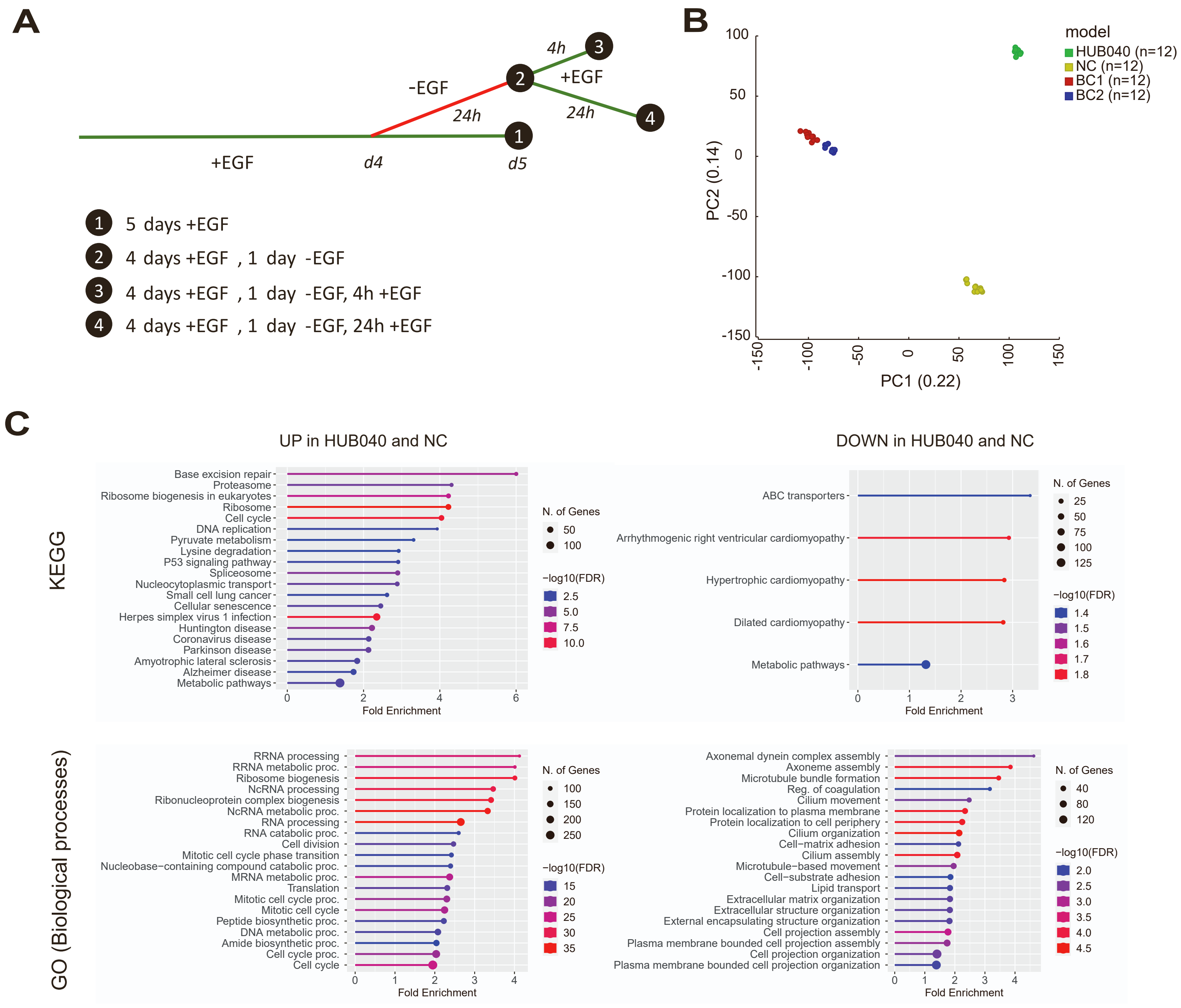

Figure S4: RNAseq analysis of BRAF-V600E mutant *versus* BRAF-corrected organoids. (A) RNAseq experimental conditions for HUB040, NC, BC1 and BC2. Of each condition three technical replicates were generated. (B) Principal component analysis of RNA-seq data. (C) Enrichment analysis of transcripts (ShinyGO) that were significantly differentially expressed between HUB040 and NC *versus* BC1 and BC2 (|log2FC| > 0.5; P value < e-10), using the KEGG, and Gene Ontology (GO) Biological Processes genesets. EGF, epidermal growth factor; FDR, false discovery rate; PC, principal component.

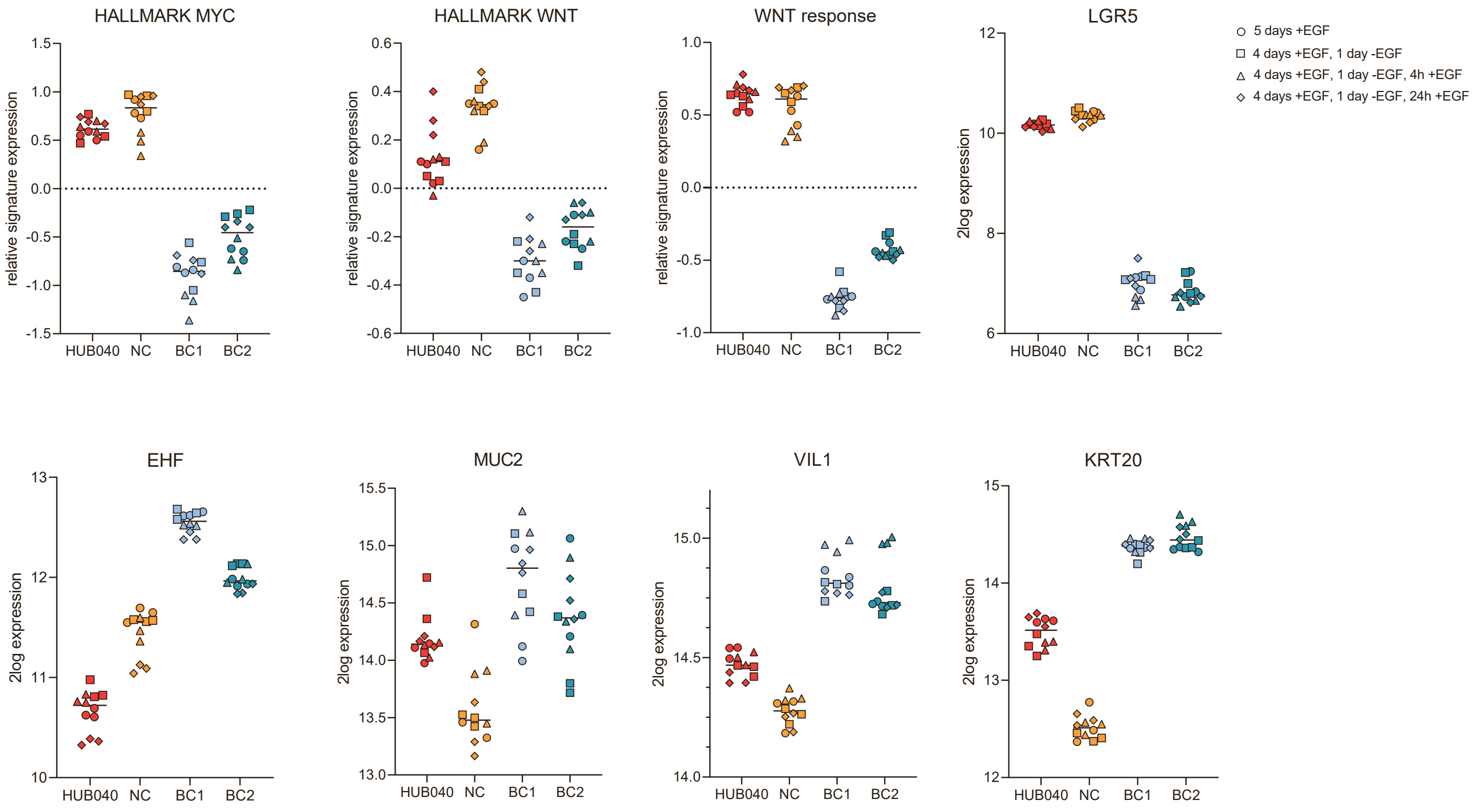

Figure S5: WNT/MYC signature and differentiation marker expression in BRAF-V600E mutant *versus* BRAF-corrected organoids. Relative signature expression (z-scores) of Hallmark MYC, Hallmark WNT, and WNT response signature (Van der Flier *et al.*) in HUB040 and NC *vs.* BC1 and BC2. 2log expression of LGR5, EHF, MUC2, VIL1, and KRT20.

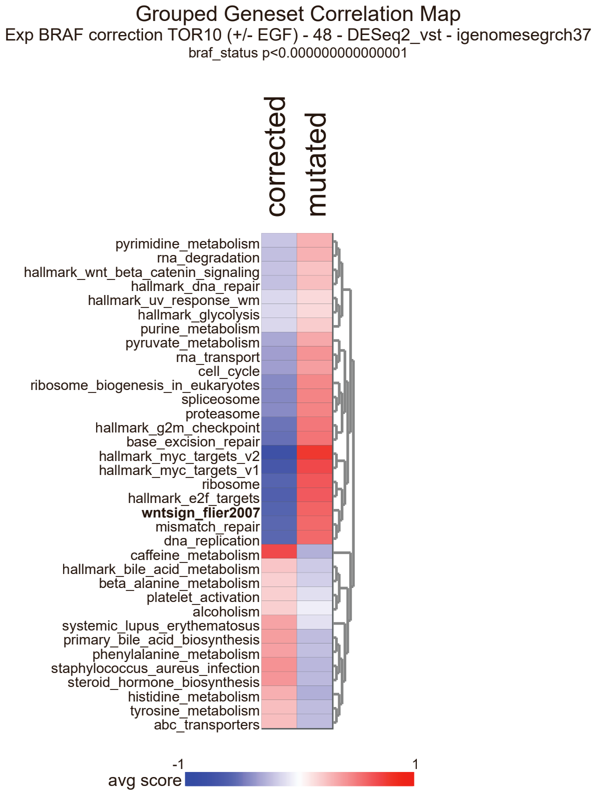

Figure S6: Log2 z-scores of all Hallmark, KEGG gene sets, and WNT response signature (Van der Flier et al.) with a P-value of < 1 x 10^{-15}.

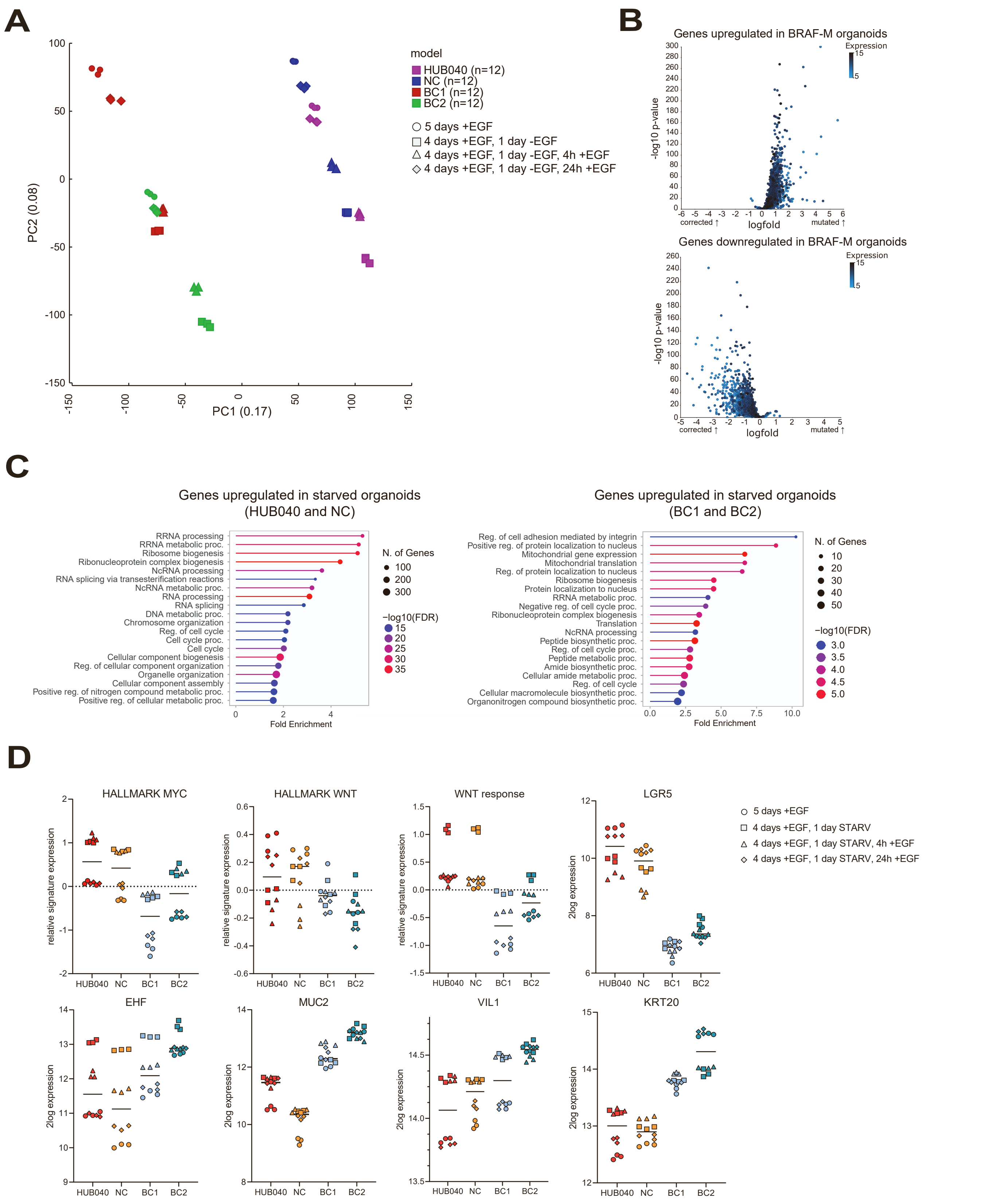

Figure S7: Transcriptomic profiles and pathway enrichment of starved organoids. (A) Principal component analysis of RNA-seq data. The analysis was conducted on organoids grown in a starvation medium composed of Advanced DMEM/F12 without the growth components required for organoid growth. (B) Expression patterns of genes previously identified as being up- or downregulated in HUB040 and NC *vs.* BC1 and BC2. These patterns were maintained even when the organoids were subjected to a more advanced starvation medium. (C) Enrichment analysis of transcripts (ShinyGO) that were significantly (P < 1 x10e-8) upregulated in starved organoids using the GO Biological Processes gene sets. (D) Relative signature expression (z-scores) of Hallmark MYC, Hallmark WNT, and WNT response signature (Van der Flier *et al.*) in HUB040 and NC *vs.* BC1 and BC2. 2log expression of LGR5, EHF, MUC2, VIL1, and KRT20.

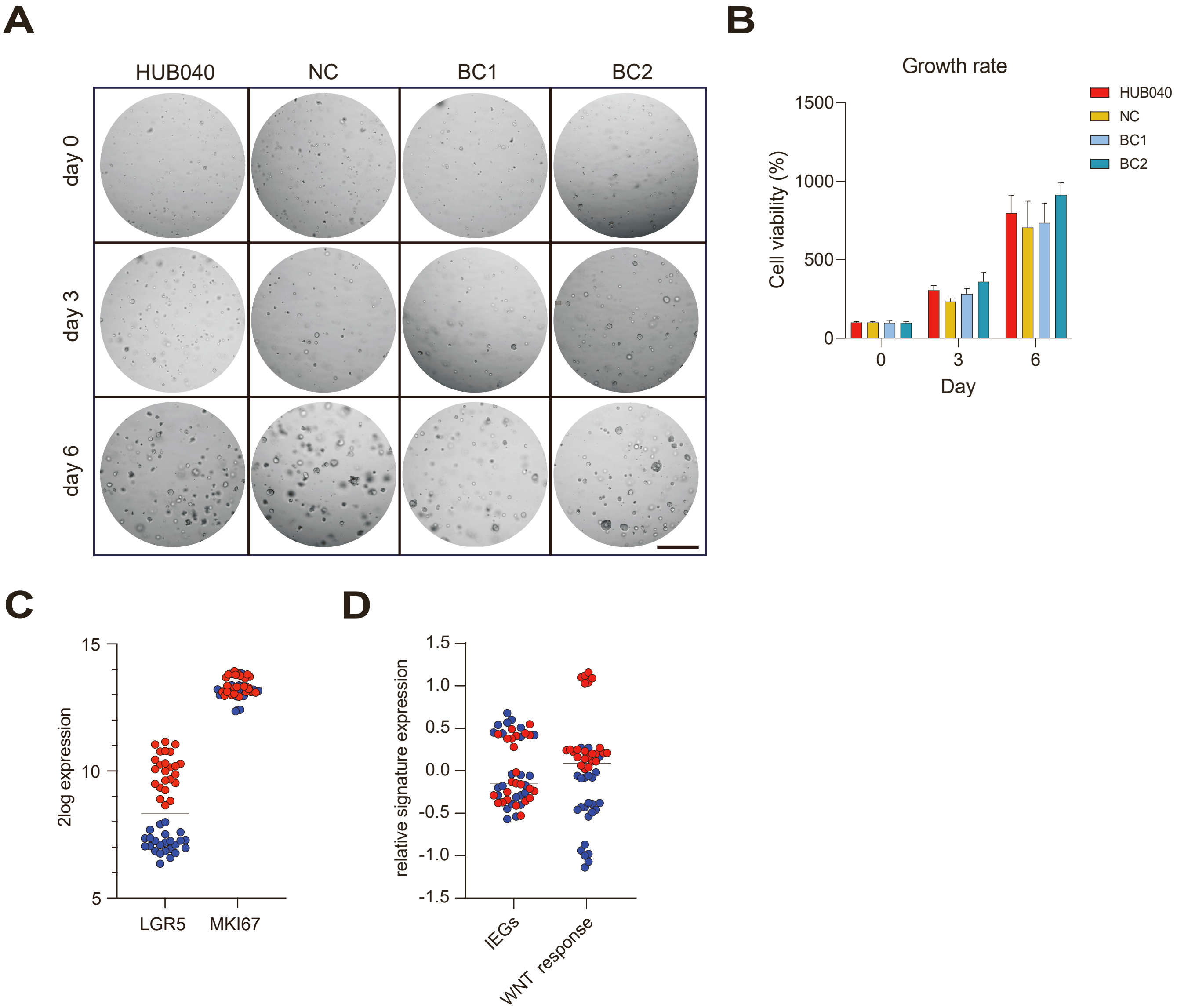

Figure S8: BC1 and BC2 organoids have similar growth rates as HUB040 and NC. (A) Organoids were plated as single cells and grown for 6 days in EGF-supplemented CRC organoid growth medium. Pictures were taken at day 0, 3 and 6. Scale bar = 400 µm. (B) Organoid viability was measured on day 0, 3 and 6 with CellTiter-Glo 3D assays. Data are presented as mean ± SD. (C) 2log expression of MKI67 and LGR5 in BRAF-V600E mutant (red) *versus* BRAF-corrected organoids (blue). (D) Relative signature expression (z-scores) of immediate early genes (Tullai *et al.*, 2007) and the WNT response signature (Van der Flier *et al.*, 2007) in BRAF-V600E mutant (red) *versus* BRAF-corrected organoids (blue).

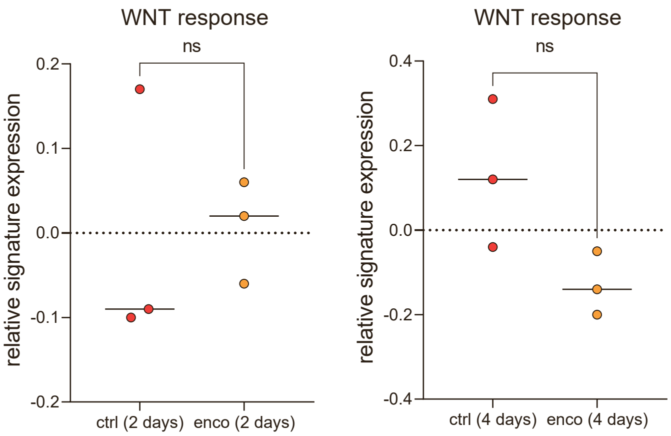

Figure S9: Relative signature expression (z-scores) of the WNT response signature (Van der Flier et al., 2007) in HUB040 organoids treated with 1 μM encorafenib for 2 or 4 days.

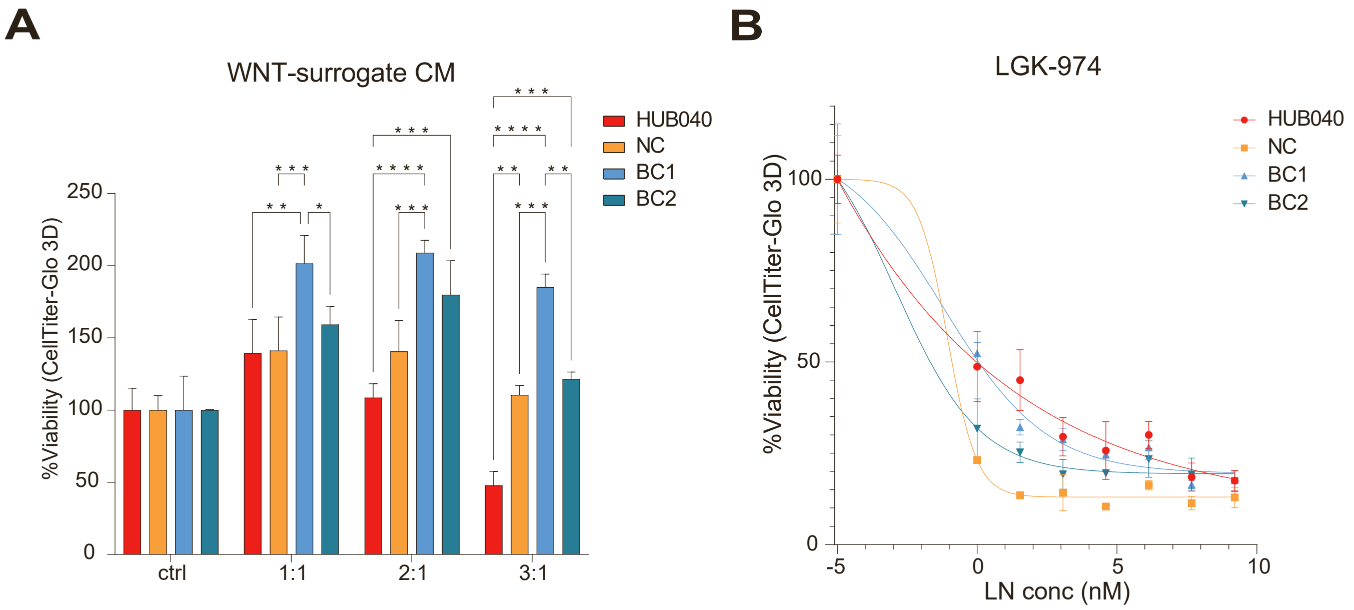

Figure S10: WNT dependency of BRAF-V600E mutant and BRAF-corrected organoids. (A) Organoids were plated as single cells and cultured with CRC organoid medium supplemented with WNT-surrogate conditioned medium at different concentrations. Organoid viability was measured with CellTiter-Glo 3D assays on day 7. (B) Organoids were treated with the porcupine inhibitor LGK-974 for 7 days after which viability was measured with CellTiter-Glo 3D assays. Data are presented as mean ± SD.

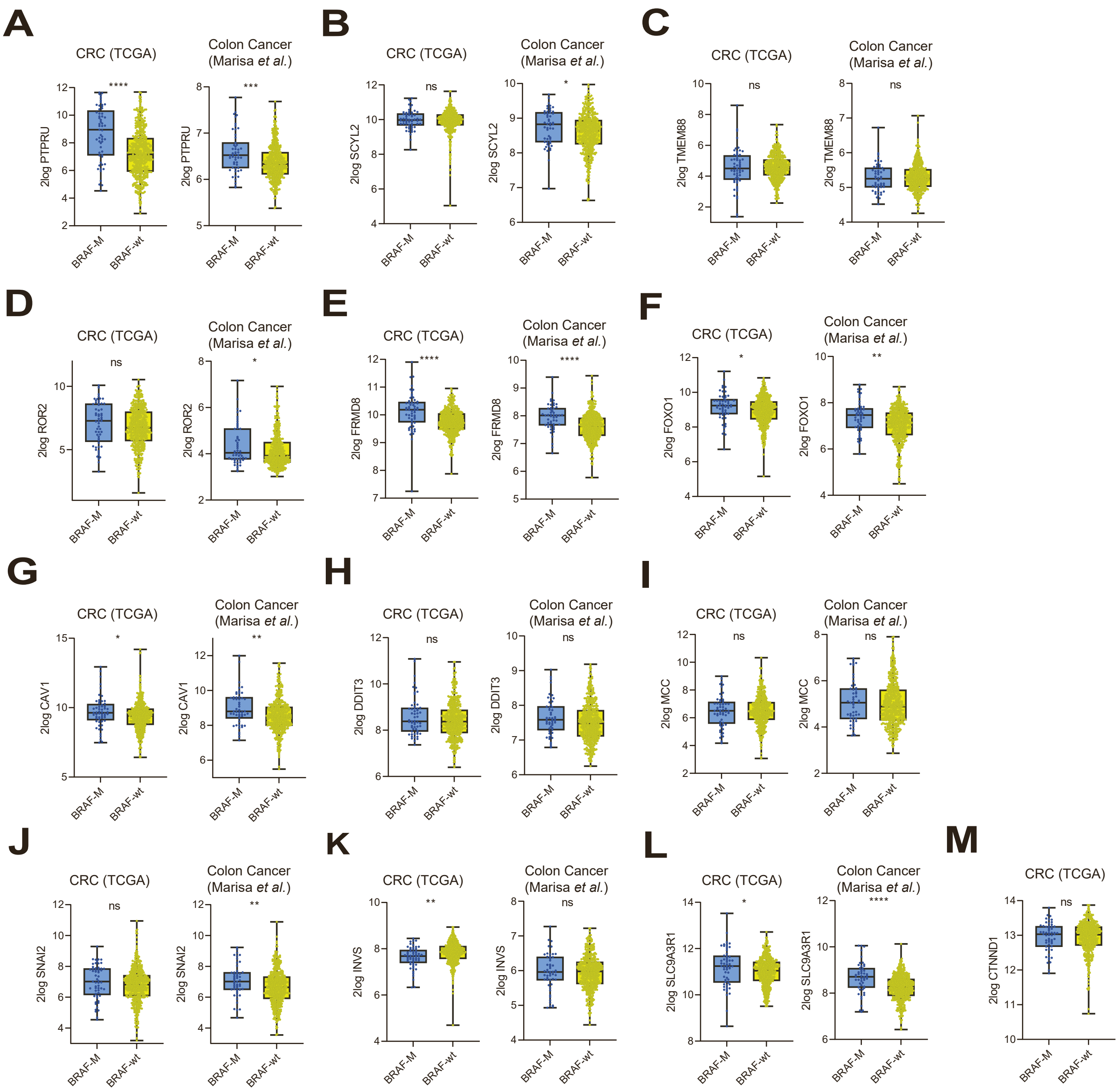

Figure S11: 2log expression of WNT antagonist genes that are transcriptionally downregulated in HUB040 and NC vs. BC1 and BC2 in the CRC TCGA dataset (BRAF-M, n=57; BRAF-wt, n=467) and colon cancer dataset Marisa et al. (BRAF-M, n=51; BRAF-wt n=461). Unpaired t-test *P < 0.05, **P < 0.01, ***P < 0.001, ****P < .0001. CRC, colorectal cancer; TCGA, The Cancer Genome Atlas.

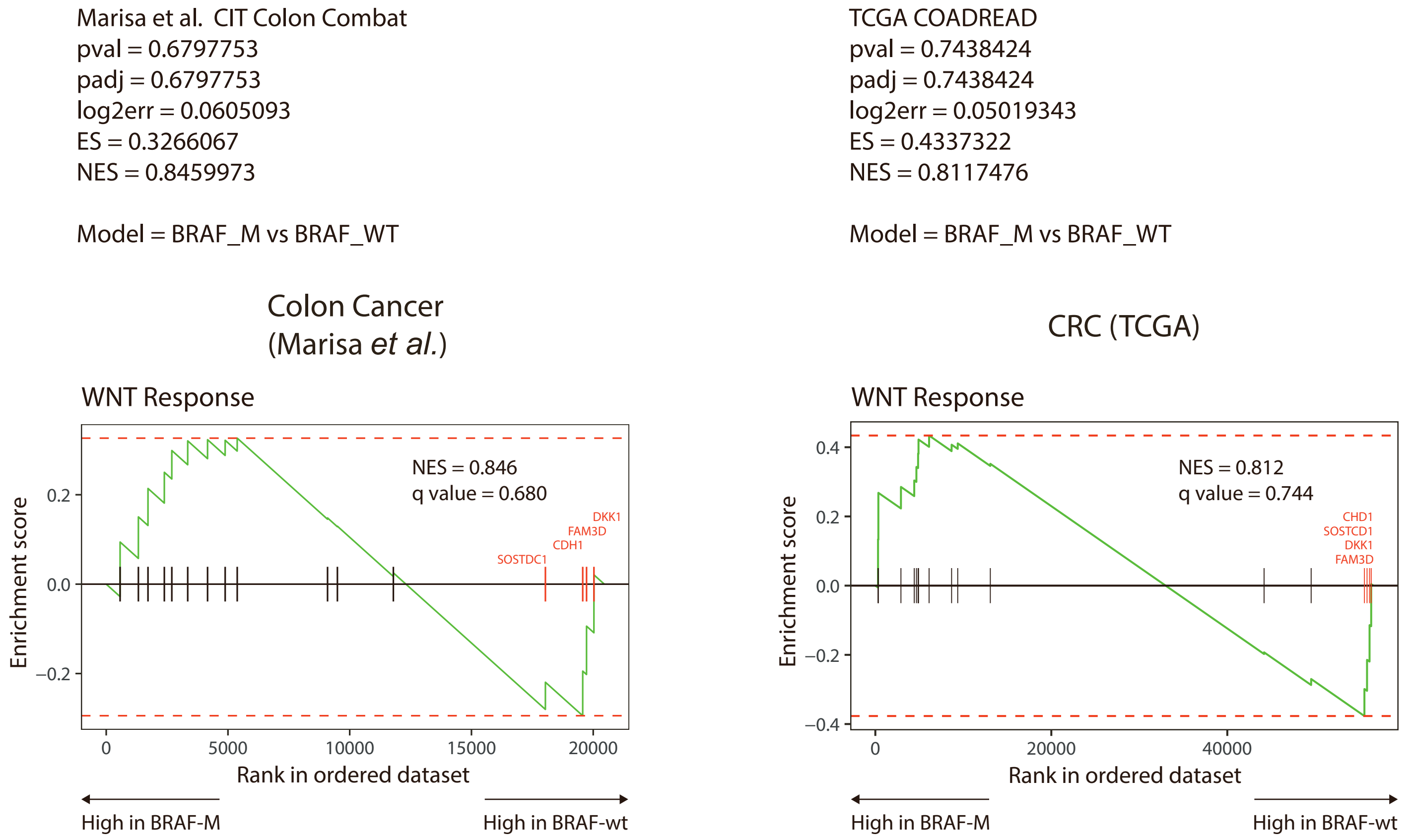

Figure S12: GSEA in the Marisa (CIT Colon Combat) and TCGA COADREAD data sets, using WNT antagonist genes that were downregulated in HUB040 and NC versus BC1 and BC2. *P*-values indicating the significance of enrichment were estimated by 1,000 permutations. CRC, colorectal cancer; ES, enrichment score; NES, normalized enrichment score; TCGA, The Cancer Genome Atlas.

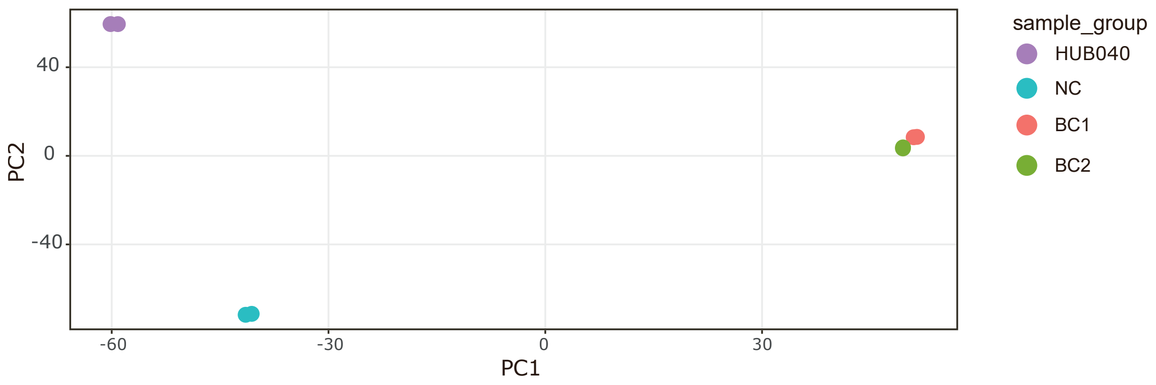

Figure S13: Principle component analysis (PCA) of DNA methylation changes between HUB040 and NC versus BC1 and BC2. Two technical replicates were generated per organoid line. PC, principal component.

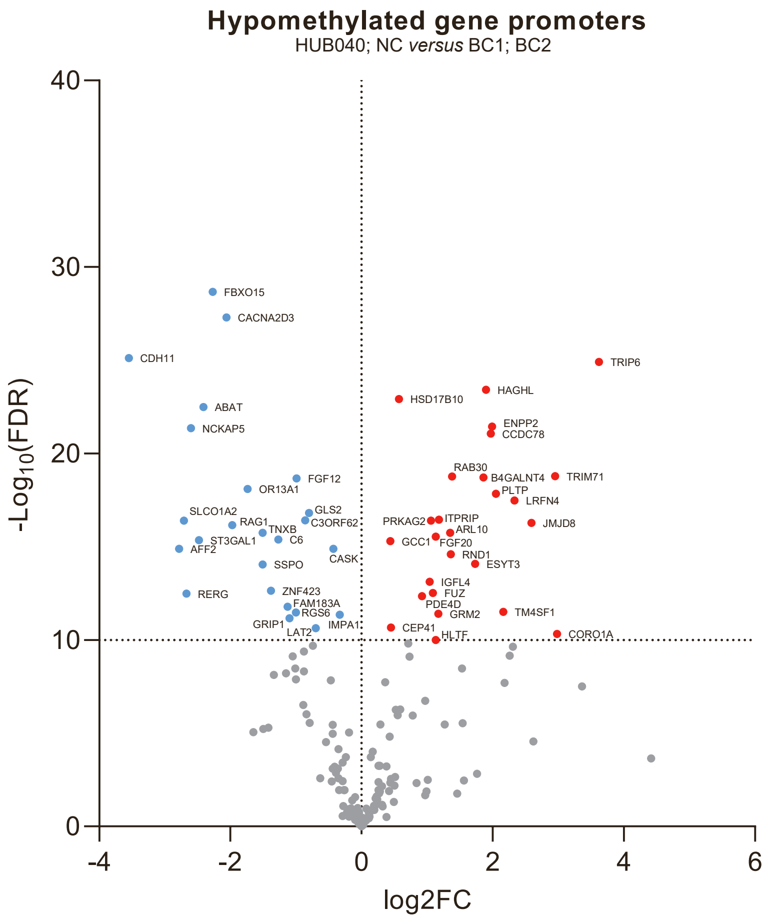

Figure S14: Volcano plot showing RNAseq expression of hypomethylated gene promoter annotated genes in HUB040 and NC versus BC1 and BC2. FC, fold change; FDR, false discovery rate.

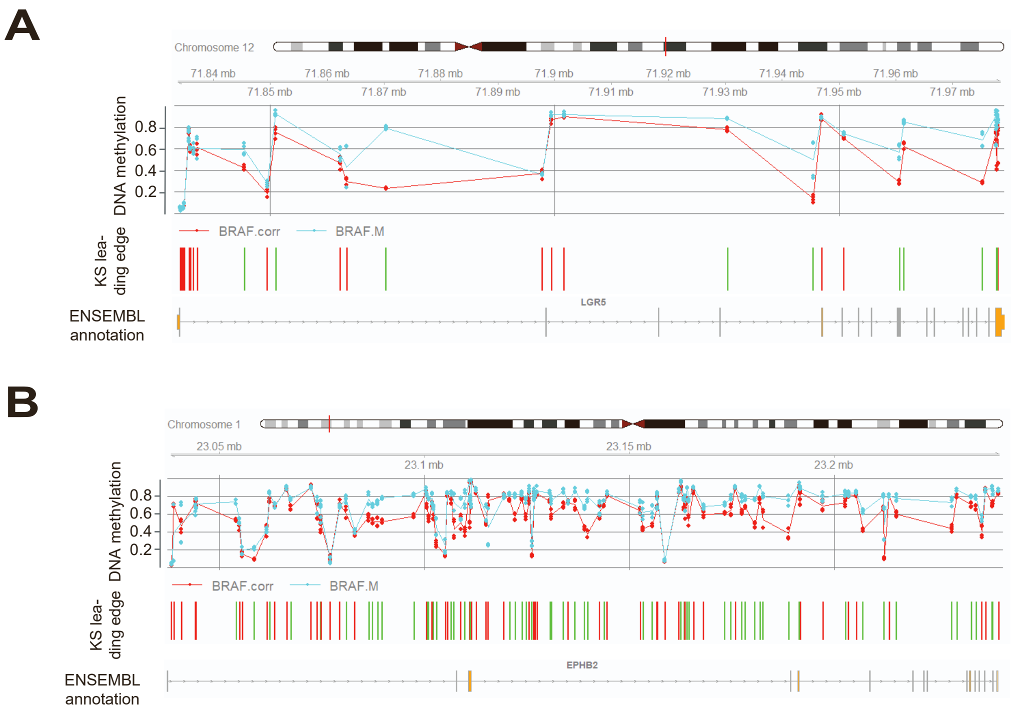

Figure S15: Gene body methylation of WNT-driven intestinal stem cell marker genes LGR5 and EPHB2. Methylation is quantified with beta values for (A) *LGR5* and (B) *EPHB2*. Each point represents the methylation of each sample and lines link the mean methylation of each group. KS leading edge panel marks with green bars CpGs contributing to the enrichment score and with red the rest of them. KS, Kolmogorov–Smirnov.

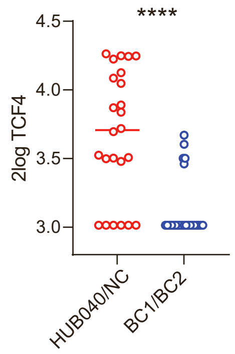

Figure S16: 2log expression of TCF4 in HUB040 and NC vs. BC1 and BC2. Unpaired *t*-test ****P < .0001.

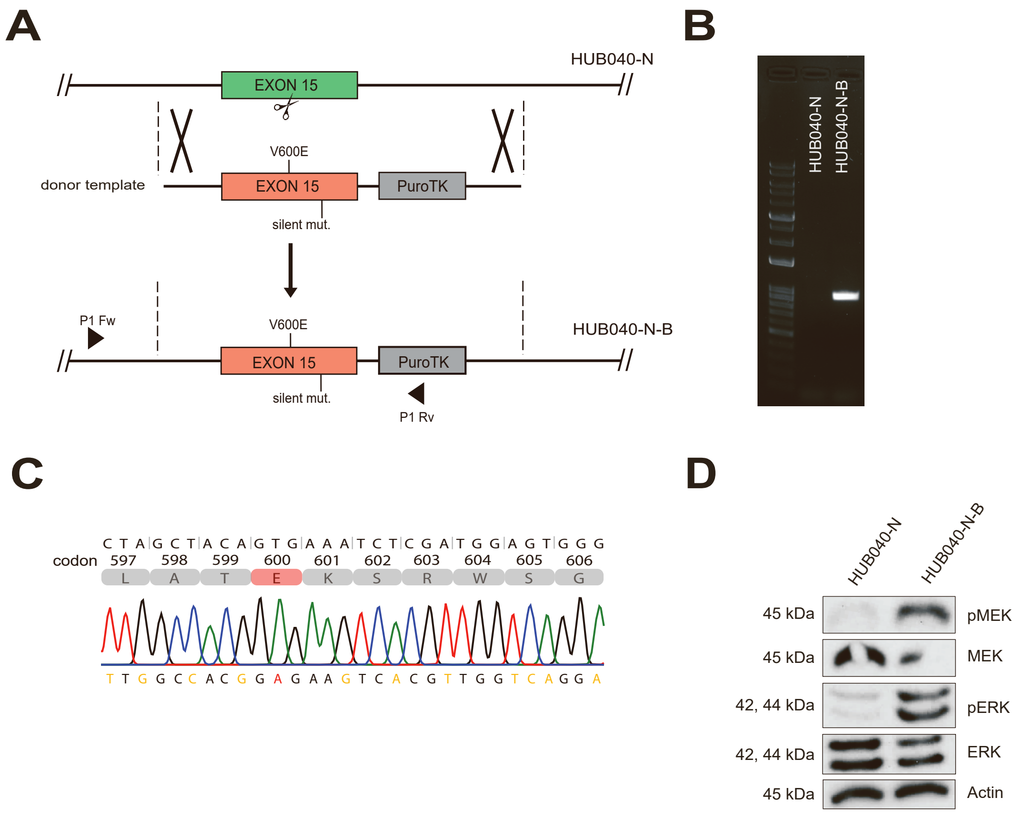

Figure S17: Knock-in of the BRAF-V600E mutation in healthy colon organoids. (A) CRISPR strategy to create a BRAF-V600E knock-in organoid. Scissors indicate the double-strand break (DSB) generated by the sgRNA targeting exon 15. A donor vector that contains the entire mutant exon 15 of the *BRAF* gene, including silent mutations, and a PuroTK selection cassette, flanked at both ends by the homology arms was used as a template to induce the homology-directed repair (HDR) mechanism after the CRISPR-Cas9-mediated DSB. Primer pair 1 was designed outside of the left homology arm to prevent detection of the donor plasmid. (B) Gel electrophesis results of the PCR product created with primer pair 1. (C) Sanger sequencing results of the PCR product created with primer pair 1. (D) Immunoblot analysis of HUB040-N and HUB040-N-B organoids.

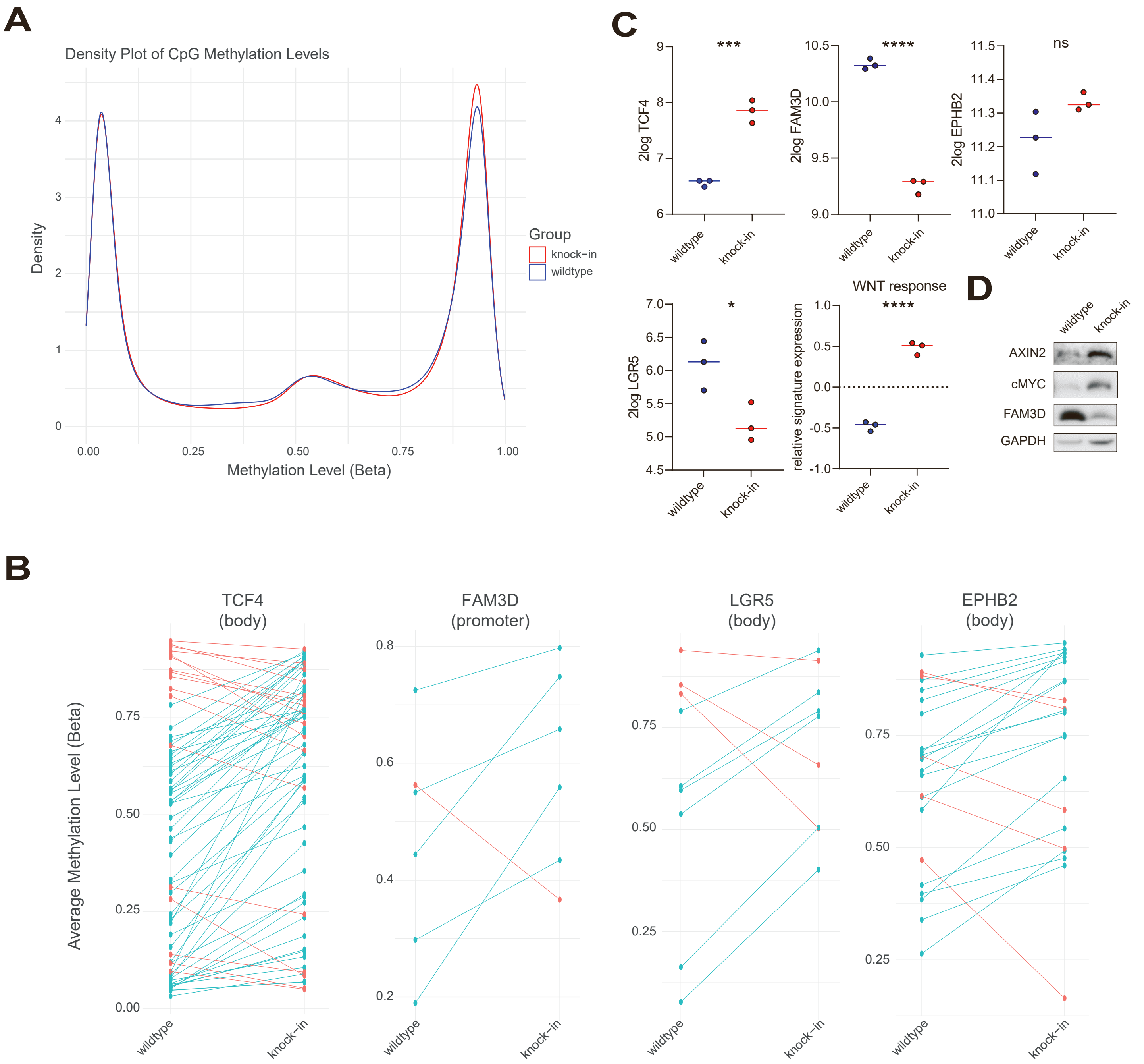

Figure S18: Introduction of the BRAF-V600E mutation in healthy colon organoids induces DNA methylation changes in key WNT-signaling genes. (A) Density plot of B-values (0 = unmethylated, 1 = fully methylated) across all probes in wildtype and BRAF-V600E knock-in organoids, with two technical replicates per PDO. Methylation data were normalized (Noob method), filtered (detection rate >95%), and analyzed with a linear model and empirical Bayes moderation. (B) Differential methylation analysis identified significant CpG sites in TCF4 (gene body), FAM3D (promoter), LGR5 (gene body), and EPHB2 (gene body). CpGs hypermethylated in knock-ins are shown in blue, and those in wildtypes in red. Difference in mean beta values represents the difference in mean methylation between wildtype BRAF and BRAF-V600E knock-in organoids. Significance was determined using adjusted p-values < 0.05 (Benjamini-Hochberg). (C) 2log expression of TCF4, FAM3D, EPHB2, and LGR5 and relative signature expression (Z-scores) of the WNT response in wildtype and BRAF-V600E knock-in organoids. (D) Western blot analysis of AXIN2, cMYC and FAM3D in wildtype and BRAF-V600E knock-in organoids. Unpaired *t*-test *P < .05, ***P < .001, ****P < .0001.

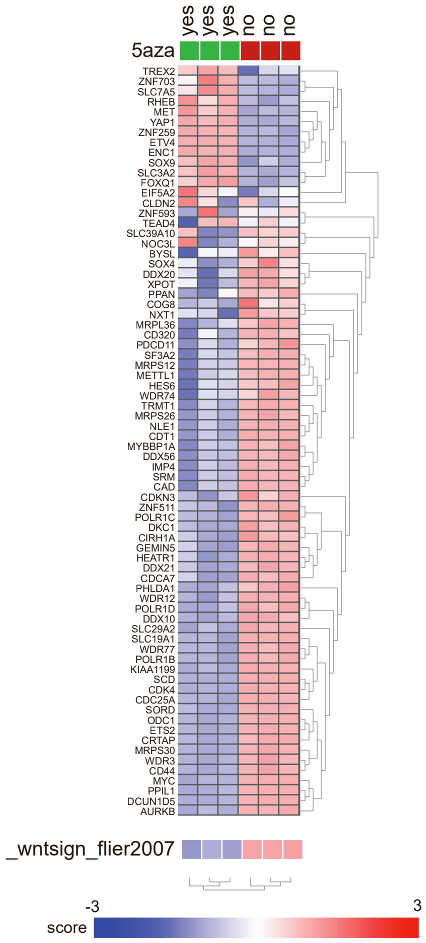

Figure S19: Heatmap of RNAseq expression z-scores of WNT target gene signature (Van Der Flier et al.) in 5-aza treated versus non-treated HUB040 organoids.

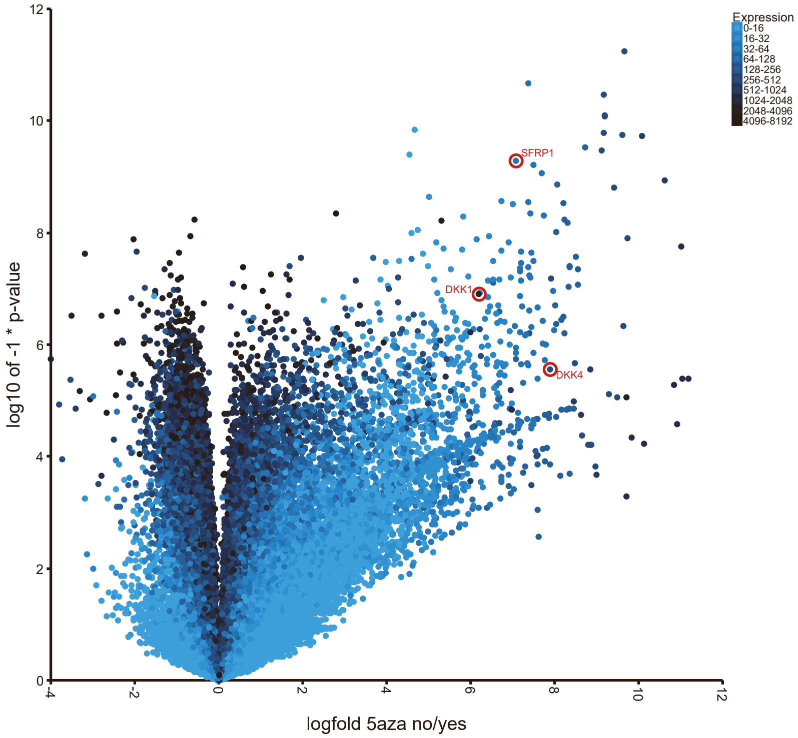

Figure S20: Volcano plot of differentially expressed genes in 5-aza treated versus non-treated HUB040 organoids.

A

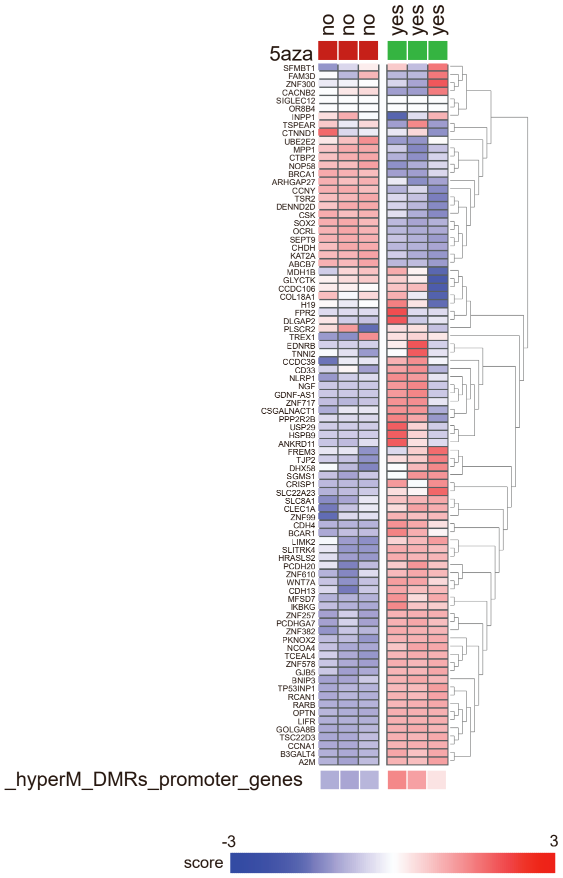

B

Gene	Gene Name
RCAN1	regulator of calcineurin 1
BNIP3	BCL2 interacting protein 3
NCOA4	nuclear receptor coactivator 4
GJB5	gap junction protein beta 5
TP53INP1	tumor protein p53 inducible nuclear protein 1
B3GALT4	beta-1,3-galactosyltransferase 4
WNT7A	Wnt family member 7A
LIFR	LIF receptor subunit alpha
IKBKG	inhibitor of nuclear factor kappa B kinase regulatory subunit gamma
ZNF610	zinc finger protein 610
OPTN	optineurin
MFSD7	major facilitator superfamily domain containing 7
HRASL2	HRAS-like suppressor 2

Figure S21: Expression effects of 5-aza treatment on genes with hypermethylated promoters in BRAF-V600E mutant organoids. (A) Heatmap of RNAseq expression z-scores of hypermethylated promoter annotated genes (found in HUB040 and NC *versus* BC1 and BC2) in 5-aza treated *versus* non-treated HUB040 organoids. (B) Hyper-methylated promoter annotated genes (found in HUB040 and NC *versus* BC1 and BC2) that are both upregulated in BC1 and BC, and in organoids treated with 5-aza.
